# Supplementary material for: Chronic Salicylate Toxicity Simulation
Source: MedEdPORTAL. 2018 Aug 17;14:10741. doi: 10.15766/mep_2374-8265.10741 (PMC6342349; doi:10.15766/mep_2374-8265.10741)
Supplement: Supplementary file 1 — A. Chronic Salicylate Toxicity Simulation Case.docx B. Chronic Salicylate Toxicity Supplemental Case Materials.ppt C. Chronic Salicylate Toxicity Questionnaire.docx D. Chronic Salicylate Toxicity Debrief.pptx E. Chronic Salicylate Toxicity Evaluation Form.doc F. Chronic Salicylate Toxicity Test.docx [file mep-14-10741-s001.zip › A._Chronic_Salicylate_Toxicity_Simulation_Case.docx]

| **Appendix A: MedEdPORTAL Simulation Case**  **SIMULATION CASE TITLE:** Chronic Salicylate Toxicity Simulation Case  AUTHORS: Mary Wittler, MD., David Masneri, DO., Jennifer Hannum, MD. | |
| --- | --- |
| **PATIENT NAME:** Mr. John Brown  **PATIENT AGE:** 63 years old  **CHIEF COMPLAINT:** Shortness of breath and wheezing | |
|  | |
| **Brief narrative description of case** | A 63 year-old-male with a history of CHF presents to the Emergency Department for evaluation of shortness of breath and wheezing. This occurs in the setting of a recent mild diarrheal illness and exacerbation of chronic back pain.  On ED arrival, the patient is hypotensive (SBP 90 mm Hg) and hypoxic (sats 90% RA). The patient is otherwise hemodynamically stable. The patient’s initial exam supports the diagnosis of decompensated heart failure, pneumonia or viral illness. This diagnosis is further supported by the patient’s CXR and laboratory evaluation. However, the clinical clue that the learner must troubleshoot to diagnose chronic salicylate toxicity is the elevated anion gap metabolic acidosis with a primary respiratory alkalosis on ABG. Further questioning of the patient reveals that the patient has being taking both Pepto-Bismol for his diarrhea and aspirin for his back pain. Initial resuscitative considerations include normal saline infusion and administration of a bicarbonate drip. The patient’s history of congestive heart failure, pulmonary edema, and kidney dysfunction limit fluid resuscitation. Consultation with nephrology for dialysis and consultation with MICU for bed placement is required. The local Poison Control Center (PCC) is available for consultation. |
| **Primary Learning Objectives** | By the end of this session, learners will be able to:   1. Identify the signs and symptoms of chronic salicylate toxicity in a simulated case 2. Interpret the arterial blood gas, and discriminate the causes of an elevated anion gap metabolic acidosis 3. Describe the limitations of salicylate concentrations in chronic salicylate toxicity 4. Identify the need for fluid resuscitation for this type of ingestion and the limitations in this simulation 5. Illustrate the utility of sodium bicarbonate administration to alkalinize the urine in this toxicity 6. Implement management goals for this poisoning and explain specific indications for management options |
| **Critical Actions** | 1. Perform a focused history and physical exam based on presentation 2. Initiate normal saline fluid resuscitation 3. Troubleshoot the acid base disorders and send a salicylate concentration 4. Consider alkalization with bicarbonate fluid; resident should acknowledge importance of potassium administration, and recognize complexity in this patient with renal failure 5. Discuss case with nephrology for hemodialysis 6. Admit the patient to the intensive care unit admission |
| **Learner Preparation** | Depending on the level of the learner and the focus of the educator, the learners may prepare for assessment of an acid-base disturbance before this simulation activity, and/or review the presentation and management of salicylate toxicity. One preparatory assignment each for the evaluation of acid-base disorders and the evaluation and management of salicylate toxicity are below:  Kelen G, Nicolaou D, Cline D. Chapter 15: Acid-Base Disorders. In Tintinalli JE, Stapczynski JS, Ma OJ (eds) Tintinalli's Emergency Medicine: A Comprehensive Study Guide. 8^th^ ed. New York, NY: McGraw-Hill; 2016.  O’Malley GF. Emergency Department Management of the Salicylate-Poisoned Patient. *Emergency Medicine Clinics of North America*. 2007;25(2):333-346. doi:[10.1016/j.emc.2007.02.012](https://doi.org/10.1016/j.emc.2007.02.012) |

| Initial Presentation | | | |
| --- | --- | --- | --- |
| **Initial vital signs** | BP 90/63 mm Hg, HR 61 beats/min, RR 24 breaths/min, Sats 90% (room air), Temp 97.2^o^ F (oral)  Weight 70 Kg | | |
| **Overall Appearance** | Upon entry to the room, the learners will see a fully dressed patient who is lying in bed and who appears to have mild labored breathing. No monitors are on the patient. | | |
| **Actors and roles in the room at case start** | Roles can vary depending on the number of participants in the simulation session and facilitator availability. This case can be implemented using a single participant and one facilitator/operator who can provide oral feedback and play additional historical or consultant roles via voice only. A nurse or EMS personnel can be used at the bedside to help deliver care to the patient, including performing medical interventions, as well as to providing ancillary support.  Only those interventions requested by the learner(s) should be performed. The main learner will act as the primary physician and do the primary evaluation of the patient to include obtaining a history, conducting a physical exam, and ordering any necessary medications or other interventions. Other learners in the scenario can serve as collaborators or observers.  A faculty instructor is present, either in person, or in the control room for simulation equipment. This faculty instructor may serve as the voice of the patient, as well as consultants including the poison control center, the nephrologist, and the intensivist. The faculty instructor may operate the simulation equipment by triggering manual changes as scripted, or allow this operation to be performed by an assistant. The faculty instructor observes the performance of the learner(s), provides feedback, and facilitates the debriefing session. | | |
| **HPI** | The patient presents with a 5 days illness of dry cough, mild shortness of breath, and wheezing. The cough is dry and nonproductive, associated with mild wheezing. The patient notes mild SOB with exertion, but none at rest. He thought his illness might be secondary to his heart or a cold, so he came in for an evaluation.  He also notes a diarrheal illness that started 4 days ago. He has had several nonbloody, nonmucoid diarrheal stools daily. The stools were initially brown, and have become dark for the past 3 days. The patient initially denies taking anything for diarrhea. The patient also had a recent exacerbation of low back pain (LBP), exacerbated it by lifting a couch about 8 days ago (no falls or other trauma). He has a history of chronic LBP; this is a similar aching pain across his entire low back that has improved. He typically takes Tylenol for pain.  (Only when asked by the learner) The patient has been taking an over-the-counter extra strength aspirin product (aspirin 500 mg per tablet), 10 tabs daily for the past week, with improvement in his back pain. Additionally, he also started taking Pepto Bismol 4 days ago for his diarrhea.  ROS (remainder of review of systems is unremarkable):  Constitutional: He denies fever, chills, weight change.  HEENT: He denies congestion, sore throat, tinnitus, or hearing changes.  Cardiovascular: He denies CP or orthopnea; he has stable 2 pillow PND. He has mild, baseline ankle and leg edema.  Pulmonary: see HPI.  Gastrointestinal: He denies abdominal pain, nausea, or vomiting.  Musculoskeletal: He denies current back pain.  Neuro: Denies numbness, weakness, bowel or bladder incontinence.  Request for old records: Recent cardiac echo (within past year) shows global hypokinesis with an EF of 20%. Recent cardiac catheterization shows no vessel disease (clean catheterization). Baseline creatinine is 1.2 | | |
| **Past Medical/Surgical History** | **Medications** | **Allergies** | **Family History** |
| CHF - Heart failure with reduced ejection fraction (HFrEF):EF 20% | Lisinopril  Furosemide  Carvedilol  OTC: Pepto Bismol and Extra strength aspirin (aspirin 500 mg per tablet) | No known allergies | None contributory |
| **Physical Examination** | | | |
| **General** | Patient is mildly tachypneic at rest, but in no acute distress. | | |
| **HEENT** | Atraumatic and normocephalic. Pupils are equal, round and reactive to light. Conjunctive are normal. Extra-ocular movements are intact with no nystagmus. Bilateral tympanic membranes are clear. Mucous membranes are dry. | | |
| **Neck** | Mild jugular venous distention; trachea midline. | | |
| **Lungs** | Mild tachypnea, but patient speaks in full sentences. Mild, end-expiratory wheezes are present in the bilateral lung fields. Rales are auscultated bilaterally throughout the lung fields. | | |
| **Cardiovascular** | Regular rate and rhythm. No murmurs, rubs, or gallops noted. | | |
| **Abdomen** | Nontender with normal bowel sounds. Abdomen is not distended and there is no rebound. | | |
| **Back** | Non-tender to palpitation along CTLS spine, NTP pelvis. No CVAT. | | |
| **Neurological** | - - 1. Patient is alert and oriented. He has no gross motor or sensory deficit with normal reflexes. | | |
| **Skin** | Warm and dry. No diaphoresis noted. | | |
| **GU** | - - 1. Rectal exam with no hemorrhoids or fissures. Good rectal tone with intact perianal sensation. Guaiac negative black stool noted. | | |
| **Extremities** | Range of motion is normal in all four extremities. 2+ pitting edema at ankles. No cyanosis. | | |

| Instructor Notes - Changes and CASE Branch Points | | |
| --- | --- | --- |
| Intervention / Time point | Change in Case | Additional Information |
| IV saline may be administered at any time point | Blood pressure will improve marginally: BP 98/62 mm Hg (MAP 74 mm Hg) | Nurse may prompt “I’ve placed an IV, do you want me to administer fluids as the patient’s blood pressure is low?” |
| Respiratory therapy may evaluate patient at any time | None | Patient has no change in respiratory status |
| Patient placed on oxygen by nasal cannula  *Scenario time = initial assessment* | Sats increase to 97% on 3 L NC | Nurse may prompt “Do you want me to administer oxygen since to patient is hypoxic?” |
| Labs and tests should be ordered to evaluate patient’s complaints  *Scenario time = 3 minutes* | None | Nurse may prompt “What blood work do you want me to send?” Or “Do you want any other imaging?” |
| Lab tests arrive  *Scenario time = 5 minutes* | Additional lab test(s) may be needed to further evaluate the elevated anion gap metabolic acidosis (aspirin concentration, lactic acid, urine and/or serum ketones) | Learners should interpret/troubleshoot initial laboratory studies. Facilitator may prompt “How can you narrow your differential for an elevated anion gap metabolic acidosis?” |
| Learner(s) will attempt to obtain further history from patient  *Scenario time = 6 minutes* | Further historical information available: patient taking OTC Pepto Bismol and aspirin | Additional history available in HPI |
| Treatment with IV sodium bicarbonate  *Scenario time = 8 minutes* | Aspirin concentration results | Facilitator can prompt “what can you administer for salicylate toxicity” or “does this patient have salicylate toxicity” |
| Resident should identify the challenge of volume resuscitation in this patient  *Scenario time = 8-9 minutes* | Consider additional options for evaluation -  POCUS Assessment: cardiac (poor LV squeeze) and IVC ( > 2cm without respiratory variation) | Facilitator can prompt “This patient has CHF with an EF of 20%. His exam and BNP suggest an element of fluid overload. How else can you assess fluid status?” |
| Discuss with Poison Control Center  *Scenario time = 10 minutes* | PCC recommends dialysis | PCC recommends dialysis on basis of history of CHF, acute renal insufficiency, pulmonary edema, and inability to give fluids |
| Discuss with nephrology for dialysis  *Scenario time = 11 minutes* | None | Facilitator can prompt “What other treatment modalities should you consider?” |
| *Admit to Medical ICU*  *Scenario time = 12 minutes* | End Scenario | Learner(s) can be debriefed |

**Ideal Scenario Flow**

- Initial stabilization should occur, including: obtain IV access, initiate gentle IVF bolus, place the patient on cardiac and pulse oximeter monitors, place the patient on supplemental oxygen
- Obtain initial HPI and exam on the patient
- Order diagnostic testing
- Troubleshoot etiology of an elevated anion gap metabolic acidosis, obtain further HPI from patient regarding aspirin ingestion
- Start IV bicarbonate drip on recognition of salicylate toxicity, consult with Poison Control
- Consult nephrology for dialysis
- Admit the patient to the ICU

The learner(s) start the case with the patient bedded in the ED. The learner(s) should initiate an evaluation of airway, breathing, and circulation, to include direction of the nurse to place the patient on a cardiac monitor and pulse oximetry, establish initial vital signs, initiate IV access, and place the patient on supplemental oxygen. The learner may or may not initial a gentle IVF bolus after consideration of the patient’s blood pressure and respiratory status. After obtaining the HPI and a complete examination, the learner(s) should order labs and testing (EKG, CXR, CBC, comprehensive metabolic panel, BNP, troponin, +/- blood gas) to evaluate the patient’s initial complaints. The learner may have respiratory therapy evaluate the patient for a breathing treatment. The learner(s) will need to further troubleshoot the elevated anion gap metabolic acidosis, and obtain additional laboratory tests as needed to narrow the differential (lactic acid, aspirin concentration, urine and/or serum ketones). The main clinical clue that suggests salicylate toxicity is the unexplained elevated anion gap metabolic acidosis with primary respiratory alkalosis. Further historical information available from the patient confirms recent salicylate use. The other primary consideration is sepsis; other possibilities include viral or bacterial pneumonia, congestive heart failure, and acute coronary disease. Other toxic ingestions that induce bradycardia with hypotension may be considered, including alpha-2 agonists, ca-channel blocker/ beta blocker, or digoxin ingestion. Depending on the level of the learner, they can be prompted to recall the utility of sodium bicarbonate infusion and indication for initiation, or get further treatment recommendations from the Poison Control Center. The Poison Control Center can confirm the suspected diagnosis of chronic salicylate toxicity, highlight that salicylate concentrations do not correlate to toxicity in chronic toxicity, and suggest the need for dialysis. Other learning opportunities in the case include how to mix a bicarbonate drip (150 mEq sodium bicarbonate in 5% Dextrose in Water (D_5_W)) to be administered for ion trapping of salicylic acid, and the importance of correcting hypokalemia to alkalinize the urine. Ultimately, secondary to this patient’s baseline HFrEF, tenuous respiratory status, and AKI, the patient needs dialysis for definitive treatment. Nephrology and ICU should be consulted prior to the end of the case. The scenario will end in 10- 15 minutes, depending on the pace of the learner.

**Anticipated Management Mistakes**

1. *Failure to appropriately troubleshoot the elevated anion gap metabolic acidosis:* Although all learners could recount a differential of elevated anion gap metabolic acidosis when prompted, some learners did not thoroughly troubleshoot the elevated anion gap metabolic acidosis by addition of other laboratory studies (lactic acid, consideration of ketosis, salicylate concentration) as a means to narrowing the differential.
2. *Failure to recognize chronic salicylate toxicity:* This was a very common mistake across all learners. This is an uncommon presentation, and the patient doesn’t present as an overdose. As such, the learners were not expecting an ingestion as the cause of presentation. The learners typically synthesized the case information, including the patient’s history and physical exam findings, the elevated anion gap metabolic acidosis and CXR findings, as the result of a CHF or pneumonia illness. Learners would try to admit the patient to the ICU before uncovering the diagnosis of chronic salicylate toxicity. At the end of this case, numerous learners stated, “I would have totally missed that.”
3. *Failure to appreciate the severity of toxicity based on the salicylate concentration:* Upon recognizing chronic salicylate toxicity, several learners failed to appreciate the severity of this presentation. Because of lack of familiarity to this type of toxicity, residents were misled by the seemingly low salicylate concentration. This is a great opportunity to educate residents that in chronic salicylate toxicity, concentrations do not correlate to toxicity.
4. *Failure to know appropriate preparation of a bicarbonate drip:* As residents heavily rely on ED pharmacists at our institution, many did not know how to prepare a bicarbonate drip. This is well within the expected knowledge base for a senior level resident, and is an opportunity for education. Indications for the initiation and discontinuation of bicarbonate infusions for salicylate toxicity are also a key learning point.
5. *Failure to arrange for dialysis:* This was less common once learners realized the patient was toxic and the concentration was ‘real.’ However, beginner learners may not appreciate that this patient has numerous indications to perform dialysis, including renal insufficiency (decreased elimination of salicylic acid), tenuous pulmonary status, and CHF.
