# Supplementary material for: Chronic Salicylate Toxicity Simulation
Source: MedEdPORTAL. 2018 Aug 17;14:10741. doi: 10.15766/mep_2374-8265.10741 (PMC6342349; doi:10.15766/mep_2374-8265.10741)
Supplement: Supplementary file 1 — A. Chronic Salicylate Toxicity Simulation Case.docx B. Chronic Salicylate Toxicity Supplemental Case Materials.ppt C. Chronic Salicylate Toxicity Questionnaire.docx D. Chronic Salicylate Toxicity Debrief.pptx E. Chronic Salicylate Toxicity Evaluation Form.doc F. Chronic Salicylate Toxicity Test.docx [file mep-14-10741-s001.zip › C._Chronic_Salicylate_Toxicity_Questionnaire.docx]

Table 1: Post Educational Event Questionnaire

|  | Disagree Strongly | Disagree | Neutral | Agree | Strongly Agree |
| --- | --- | --- | --- | --- | --- |
| Overall, I am satisfactied with this educational event |  |  |  |  |  |
| I am better equipped to identify the signs and symptoms of a chronic salicylate intoxication |  |  |  |  |  |
| I am more confident in my ability to critically evaluate causes of acid-base disturbances |  |  |  |  |  |
| I have better understanding of the treatment goals for this poisoning and specific indications for management options |  |  |  |  |  |
| This event enhanced my knowledge of salicylate toxicity |  |  |  |  |  |
| The debrief was effective in presenting the educational objectives |  |  |  |  |  |
