# Supplementary material for: Chronic Salicylate Toxicity Simulation
Source: MedEdPORTAL. 2018 Aug 17;14:10741. doi: 10.15766/mep_2374-8265.10741 (PMC6342349; doi:10.15766/mep_2374-8265.10741)
Supplement: Supplementary file 1 — A. Chronic Salicylate Toxicity Simulation Case.docx B. Chronic Salicylate Toxicity Supplemental Case Materials.ppt C. Chronic Salicylate Toxicity Questionnaire.docx D. Chronic Salicylate Toxicity Debrief.pptx E. Chronic Salicylate Toxicity Evaluation Form.doc F. Chronic Salicylate Toxicity Test.docx [file mep-14-10741-s001.zip › D._Chronic_Salicylate_Toxicity_Debrief.pptx]

## Slide 1
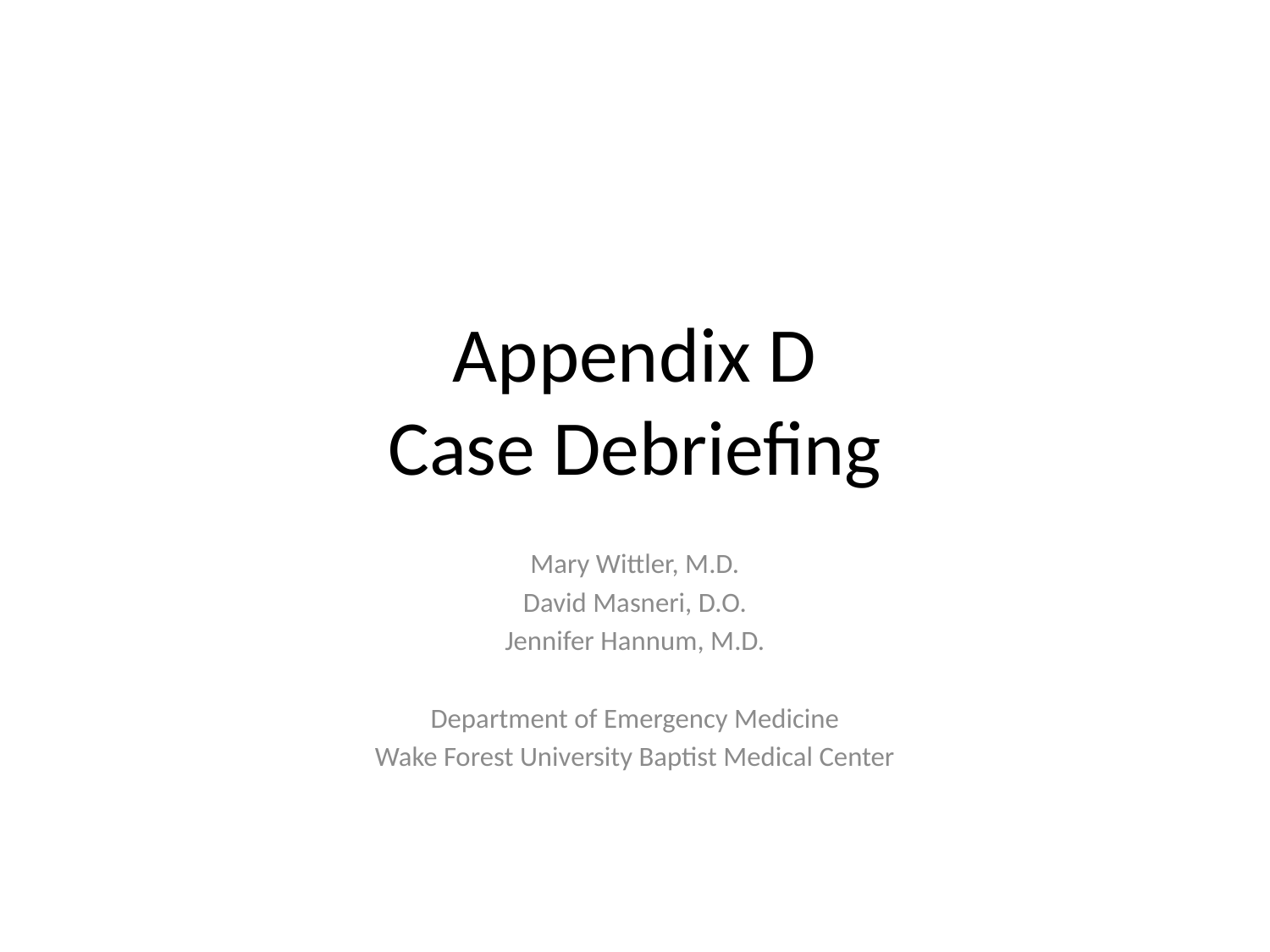

# Appendix D Case Debriefing
Mary Wittler, M.D.
David Masneri, D.O.
Jennifer Hannum, M.D.
Department of Emergency Medicine
Wake Forest University Baptist Medical Center

## Slide 2
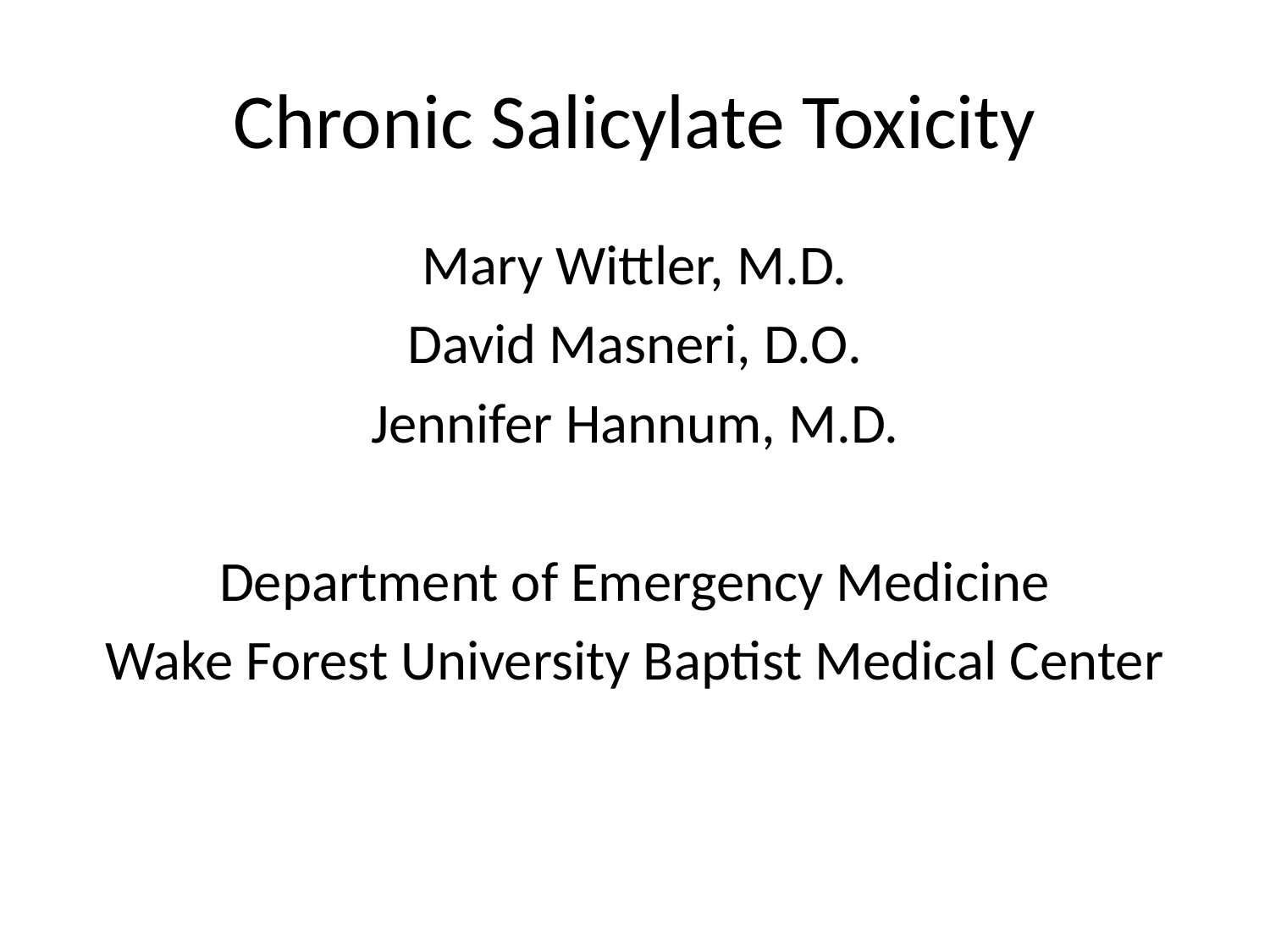

# Chronic Salicylate Toxicity
Mary Wittler, M.D.
David Masneri, D.O.
Jennifer Hannum, M.D.
Department of Emergency Medicine
Wake Forest University Baptist Medical Center

## Slide 3
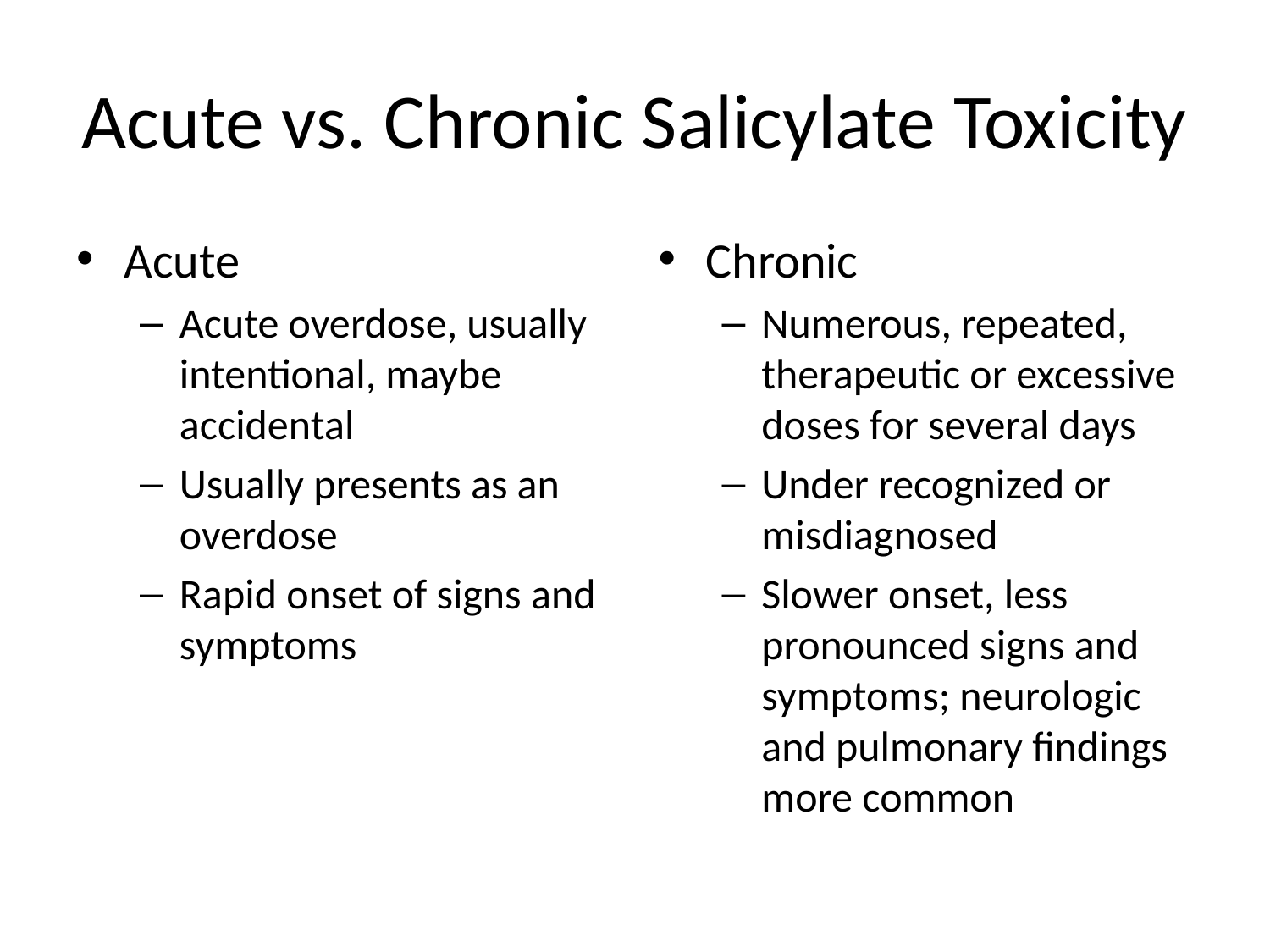

# Acute vs. Chronic Salicylate Toxicity
Acute
Acute overdose, usually intentional, maybe accidental
Usually presents as an overdose
Rapid onset of signs and symptoms
Chronic
Numerous, repeated, therapeutic or excessive doses for several days
Under recognized or misdiagnosed
Slower onset, less pronounced signs and symptoms; neurologic and pulmonary findings more common

## Slide 4
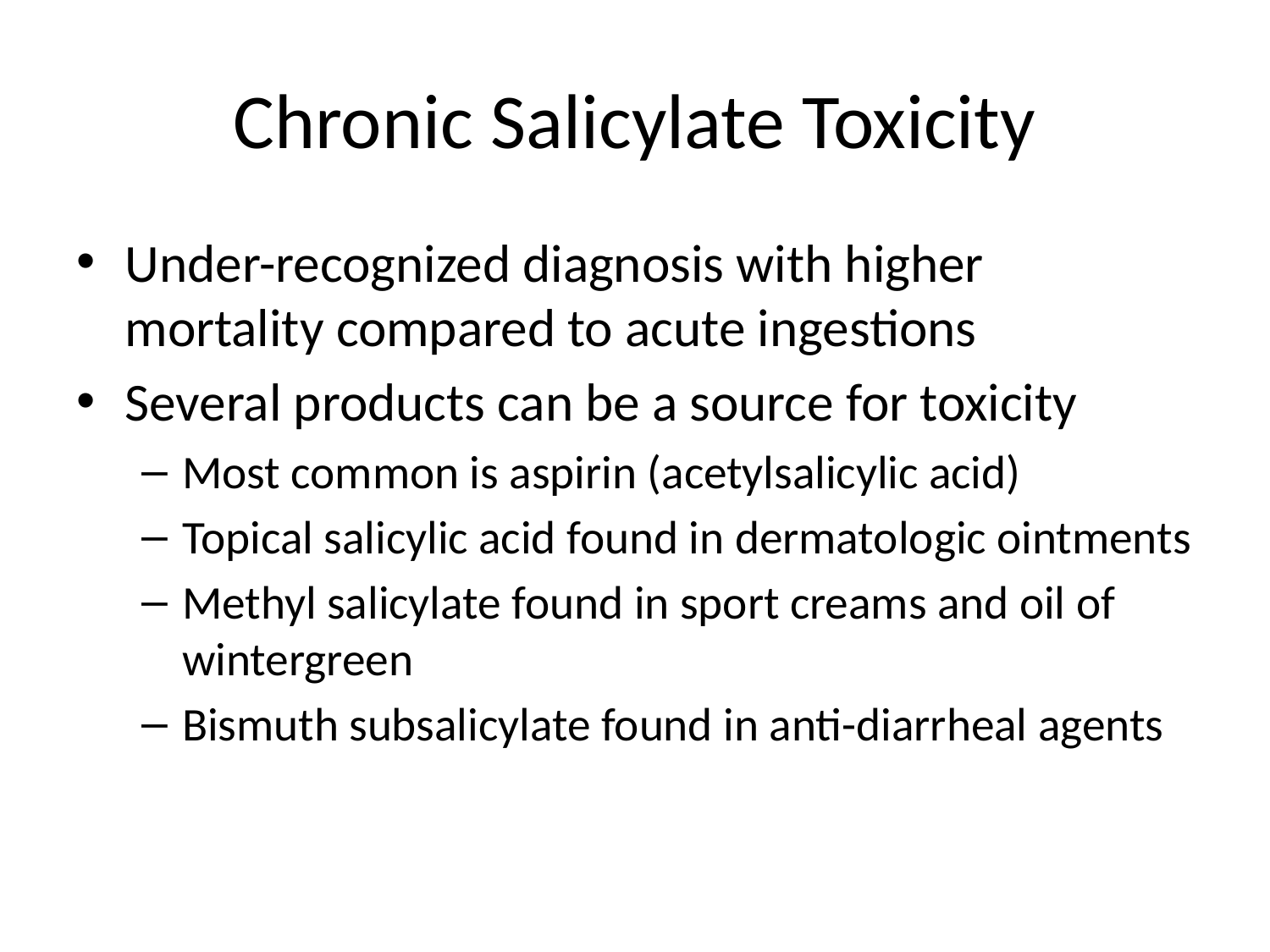

# Chronic Salicylate Toxicity
Under-recognized diagnosis with higher mortality compared to acute ingestions
Several products can be a source for toxicity
Most common is aspirin (acetylsalicylic acid)
Topical salicylic acid found in dermatologic ointments
Methyl salicylate found in sport creams and oil of wintergreen
Bismuth subsalicylate found in anti-diarrheal agents

## Slide 5
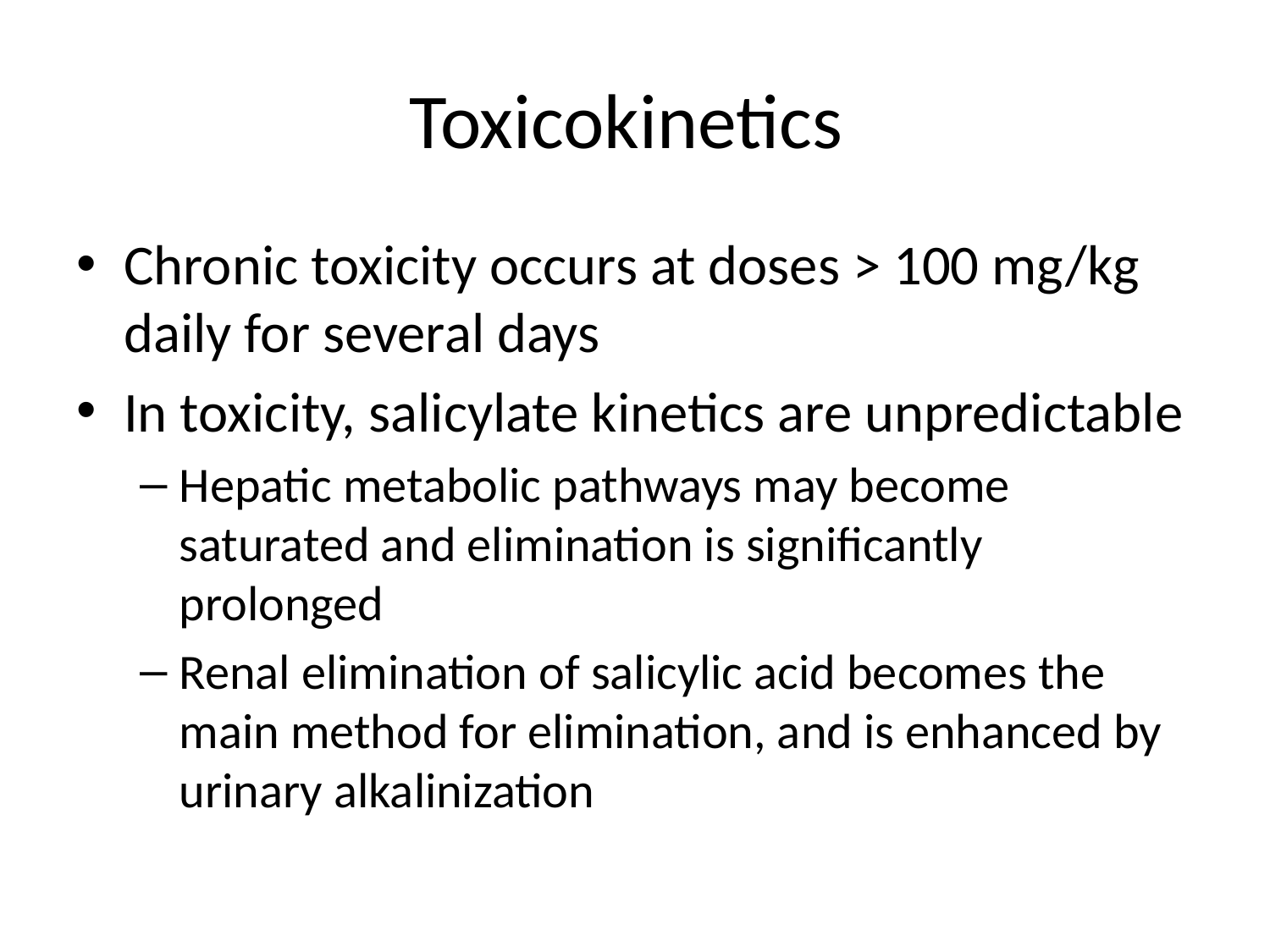

# Toxicokinetics
Chronic toxicity occurs at doses > 100 mg/kg daily for several days
In toxicity, salicylate kinetics are unpredictable
Hepatic metabolic pathways may become saturated and elimination is significantly prolonged
Renal elimination of salicylic acid becomes the main method for elimination, and is enhanced by urinary alkalinization

## Slide 6
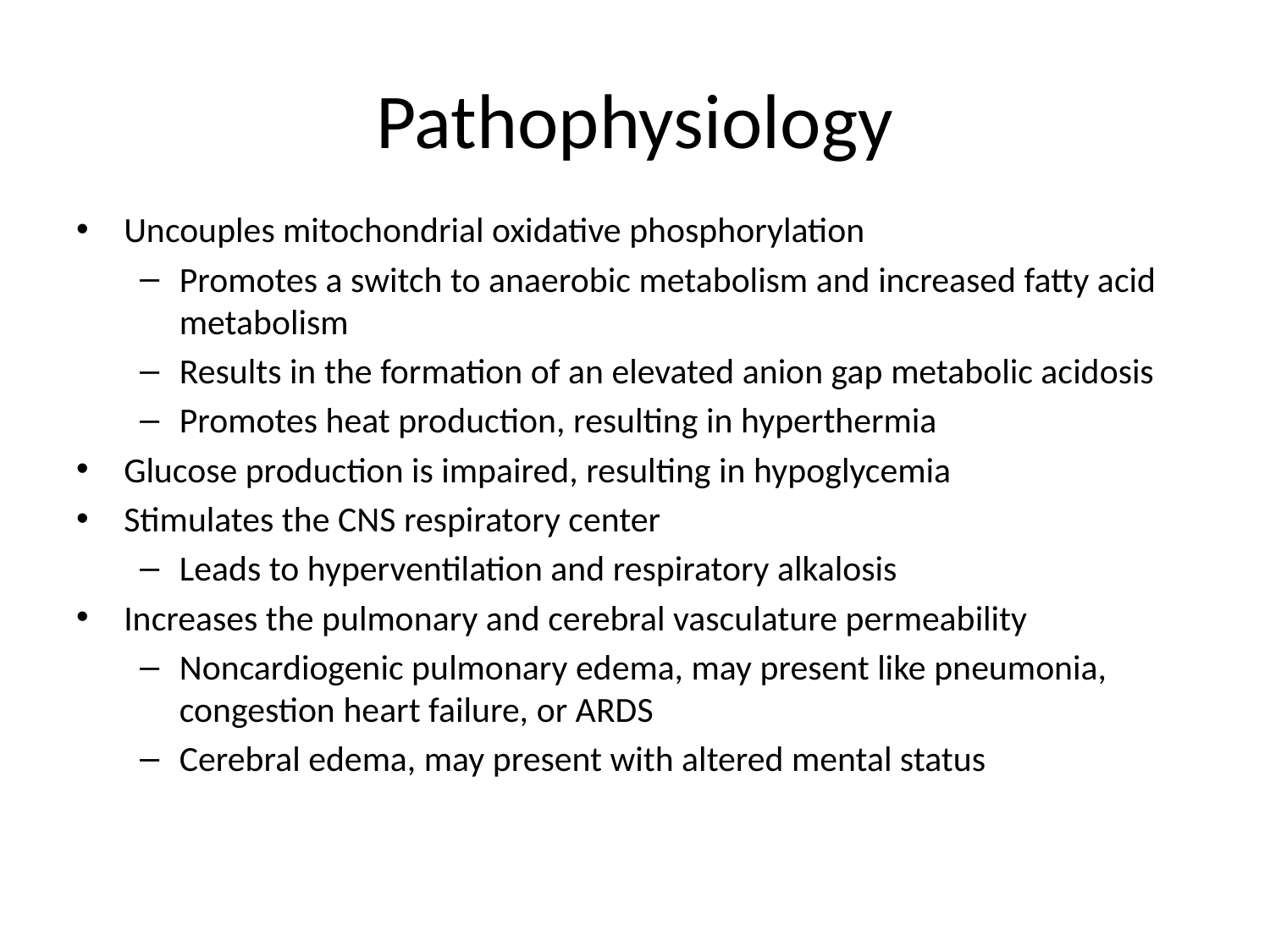

# Pathophysiology
Uncouples mitochondrial oxidative phosphorylation
Promotes a switch to anaerobic metabolism and increased fatty acid metabolism
Results in the formation of an elevated anion gap metabolic acidosis
Promotes heat production, resulting in hyperthermia
Glucose production is impaired, resulting in hypoglycemia
Stimulates the CNS respiratory center
Leads to hyperventilation and respiratory alkalosis
Increases the pulmonary and cerebral vasculature permeability
Noncardiogenic pulmonary edema, may present like pneumonia, congestion heart failure, or ARDS
Cerebral edema, may present with altered mental status

## Slide 7
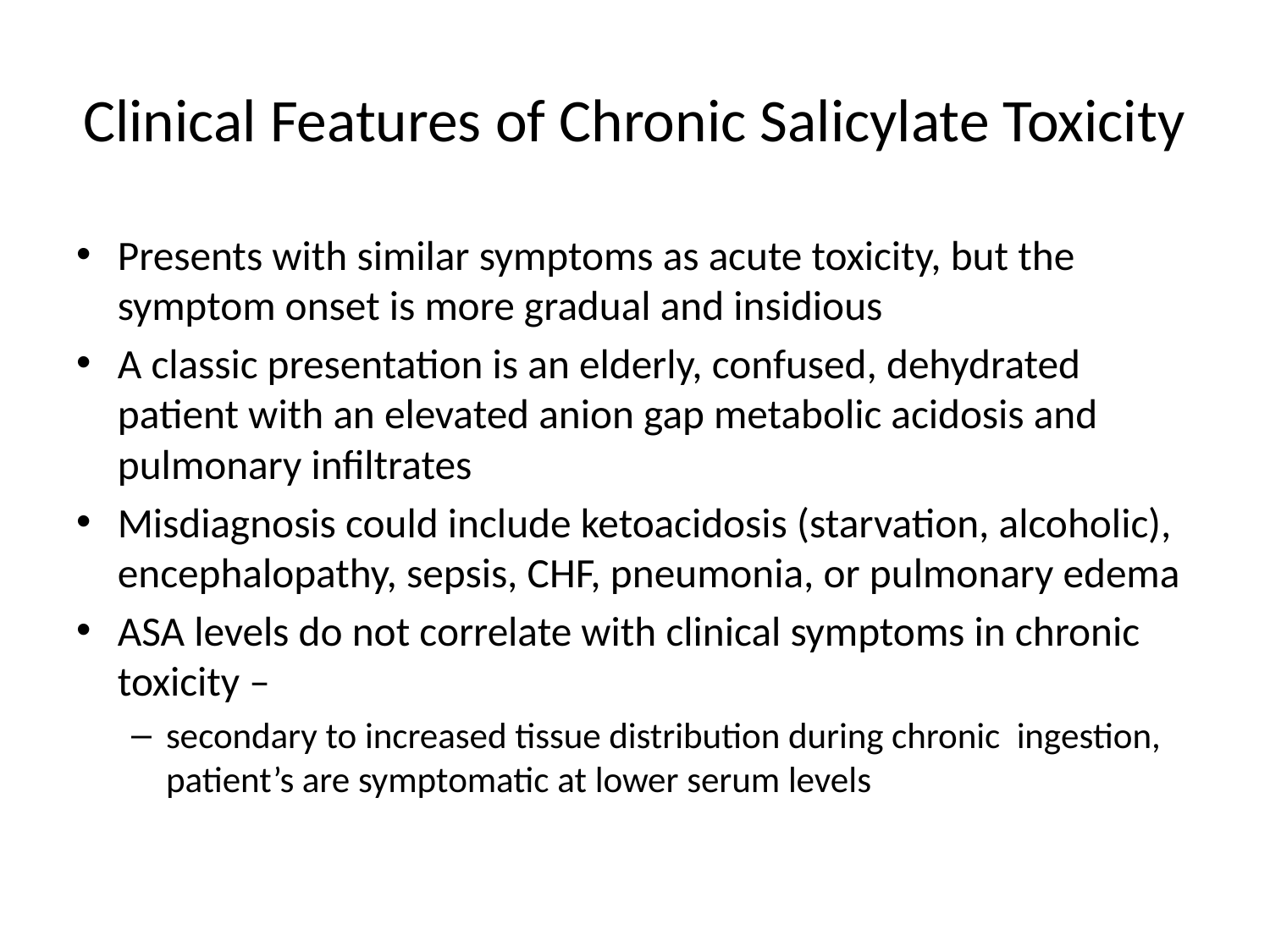

# Clinical Features of Chronic Salicylate Toxicity
Presents with similar symptoms as acute toxicity, but the symptom onset is more gradual and insidious
A classic presentation is an elderly, confused, dehydrated patient with an elevated anion gap metabolic acidosis and pulmonary infiltrates
Misdiagnosis could include ketoacidosis (starvation, alcoholic), encephalopathy, sepsis, CHF, pneumonia, or pulmonary edema
ASA levels do not correlate with clinical symptoms in chronic toxicity –
secondary to increased tissue distribution during chronic ingestion, patient’s are symptomatic at lower serum levels

## Slide 8
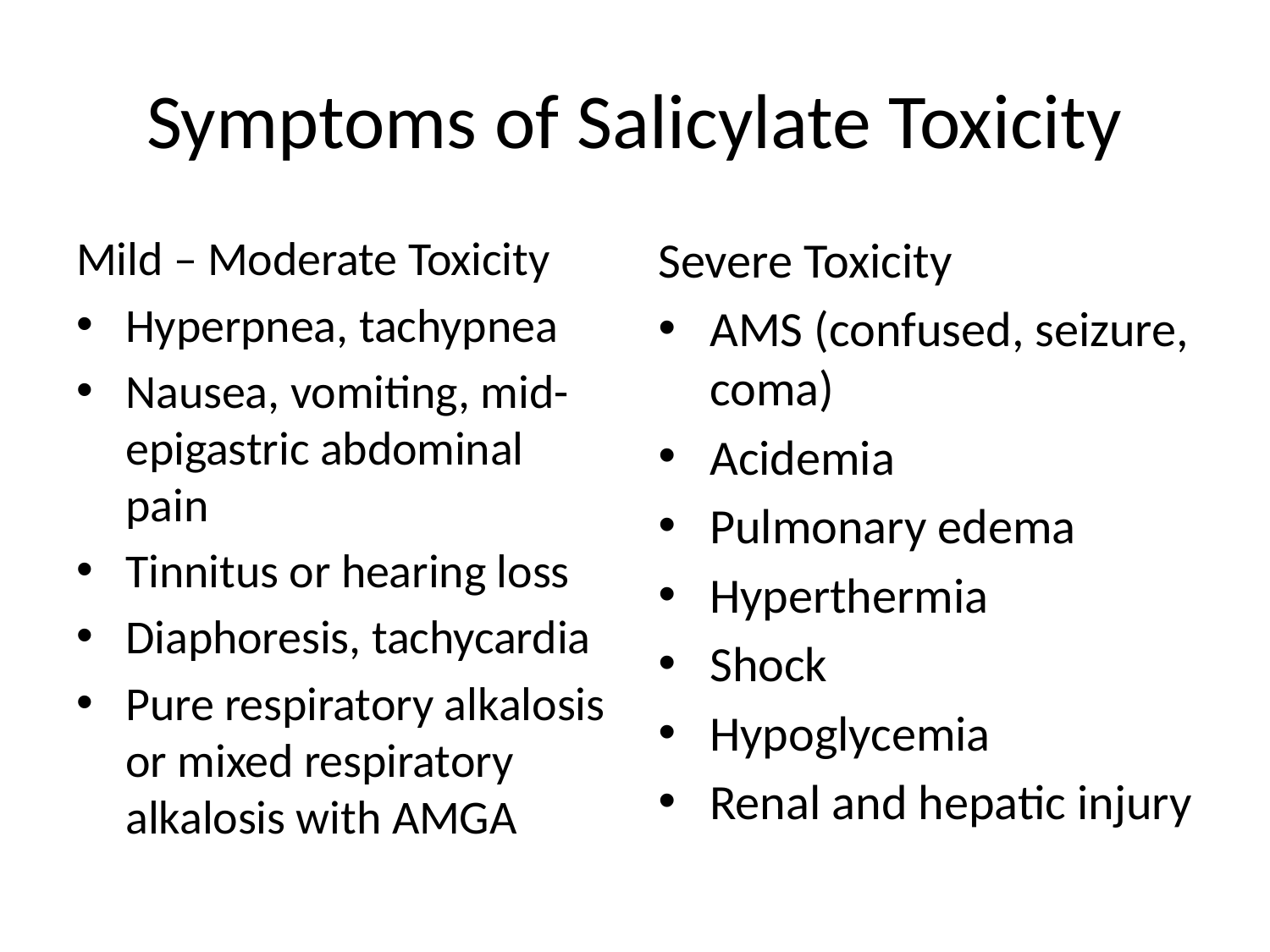

# Symptoms of Salicylate Toxicity
Mild – Moderate Toxicity
Hyperpnea, tachypnea
Nausea, vomiting, mid-epigastric abdominal pain
Tinnitus or hearing loss
Diaphoresis, tachycardia
Pure respiratory alkalosis or mixed respiratory alkalosis with AMGA
Severe Toxicity
AMS (confused, seizure, coma)
Acidemia
Pulmonary edema
Hyperthermia
Shock
Hypoglycemia
Renal and hepatic injury

## Slide 9
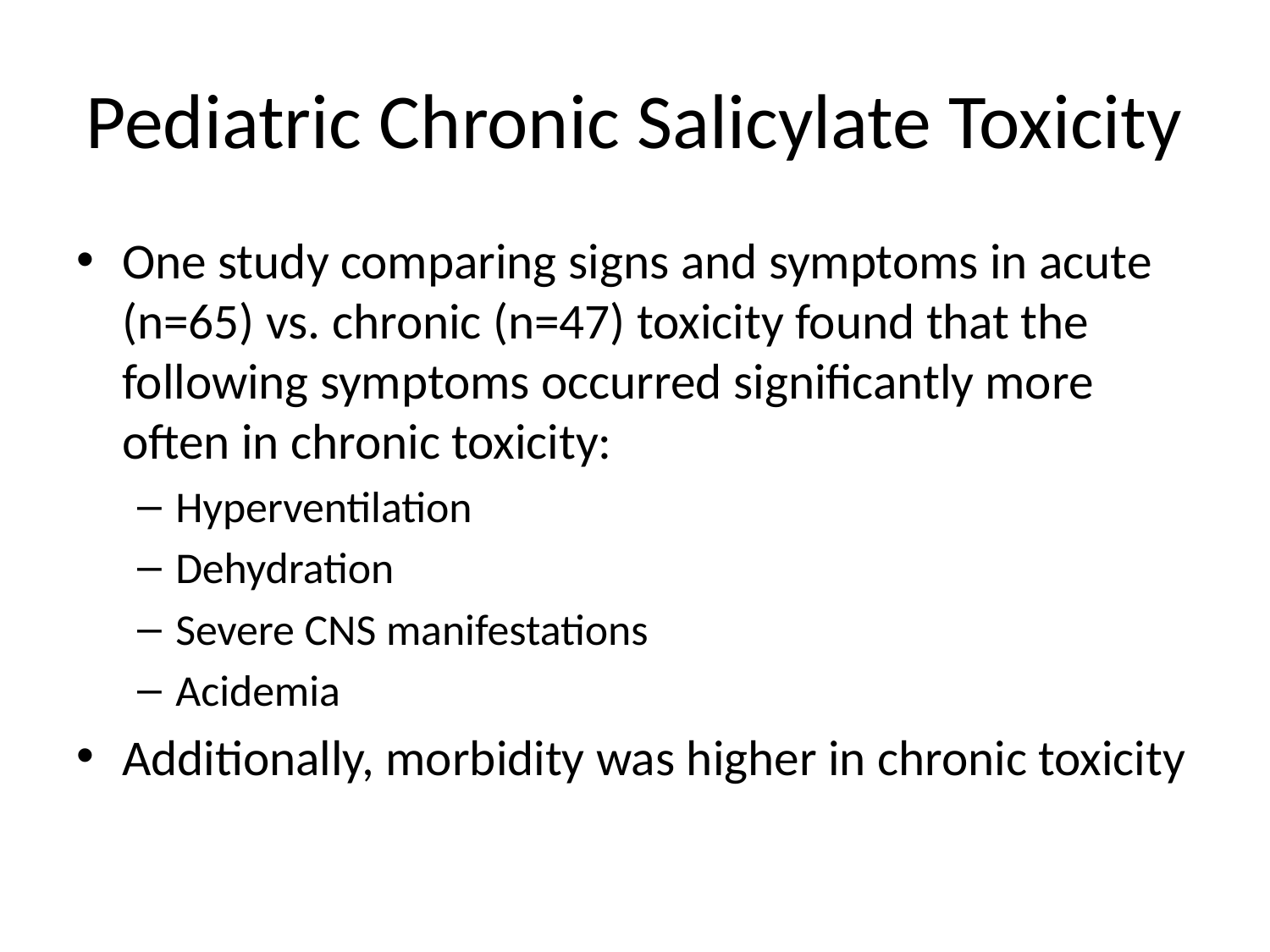

# Pediatric Chronic Salicylate Toxicity
One study comparing signs and symptoms in acute (n=65) vs. chronic (n=47) toxicity found that the following symptoms occurred significantly more often in chronic toxicity:
Hyperventilation
Dehydration
Severe CNS manifestations
Acidemia
Additionally, morbidity was higher in chronic toxicity

## Slide 10
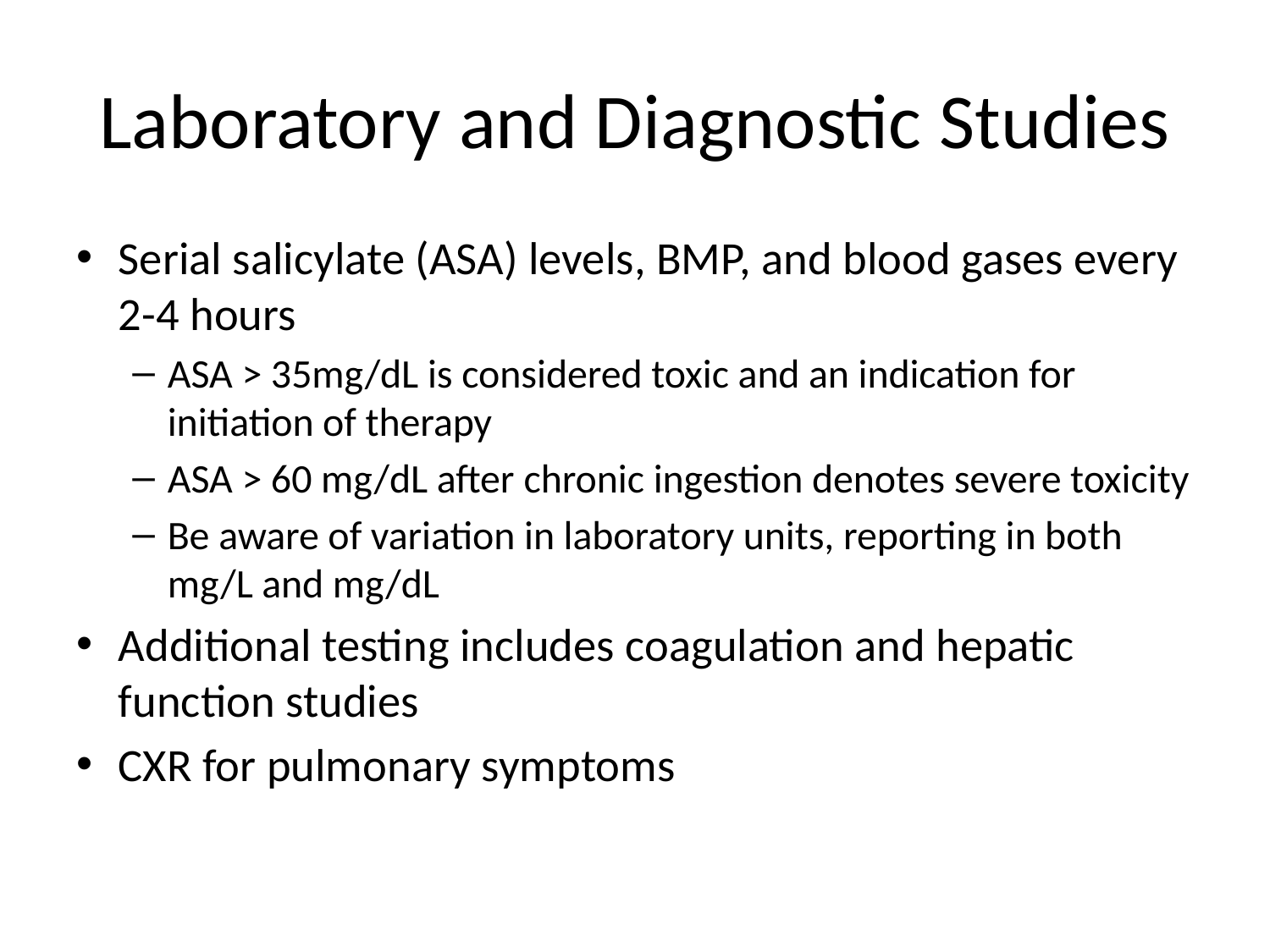

# Laboratory and Diagnostic Studies
Serial salicylate (ASA) levels, BMP, and blood gases every 2-4 hours
ASA > 35mg/dL is considered toxic and an indication for initiation of therapy
ASA > 60 mg/dL after chronic ingestion denotes severe toxicity
Be aware of variation in laboratory units, reporting in both mg/L and mg/dL
Additional testing includes coagulation and hepatic function studies
CXR for pulmonary symptoms

## Slide 11
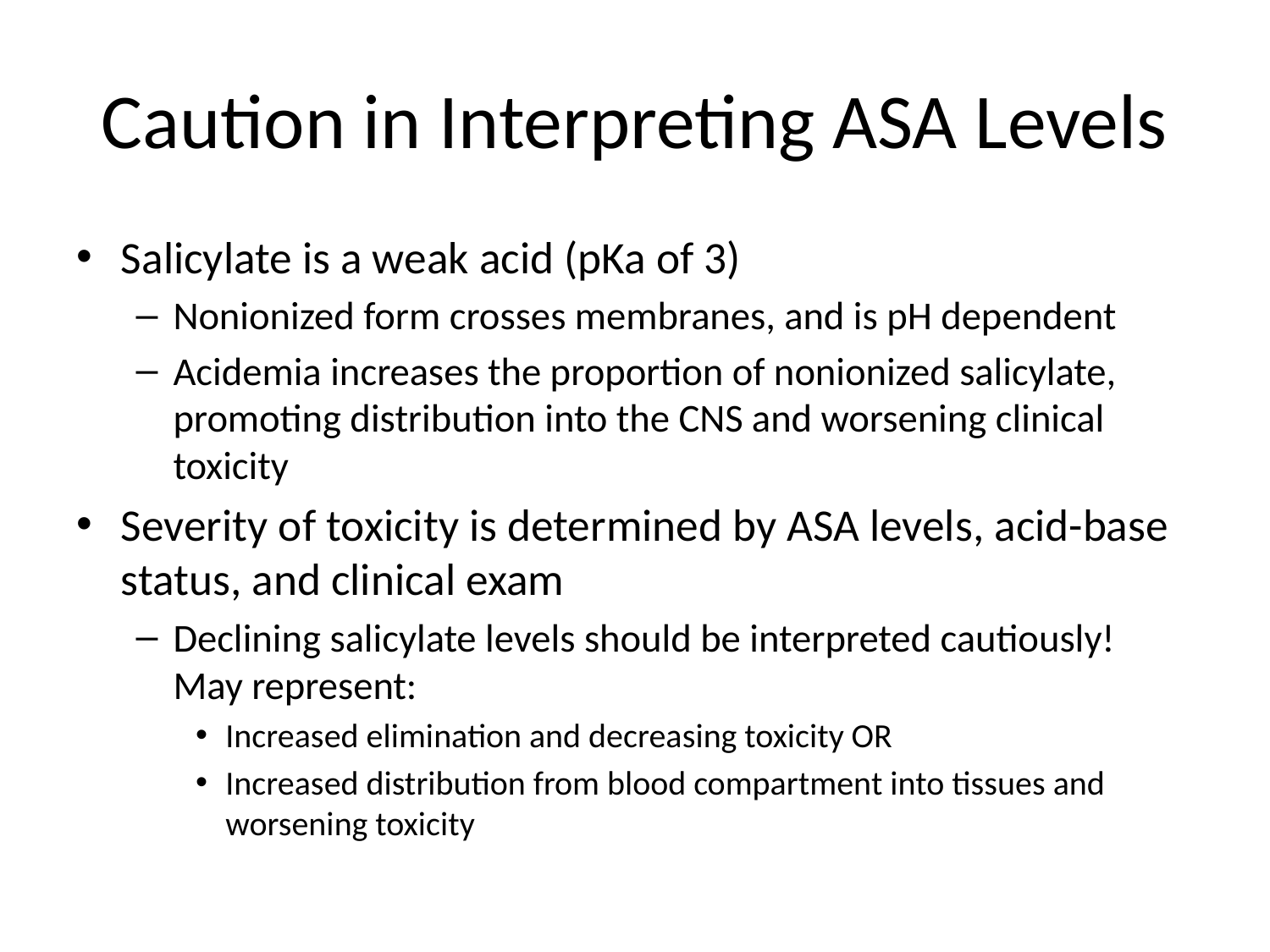

# Caution in Interpreting ASA Levels
Salicylate is a weak acid (pKa of 3)
Nonionized form crosses membranes, and is pH dependent
Acidemia increases the proportion of nonionized salicylate, promoting distribution into the CNS and worsening clinical toxicity
Severity of toxicity is determined by ASA levels, acid-base status, and clinical exam
Declining salicylate levels should be interpreted cautiously! May represent:
Increased elimination and decreasing toxicity OR
Increased distribution from blood compartment into tissues and worsening toxicity

## Slide 12
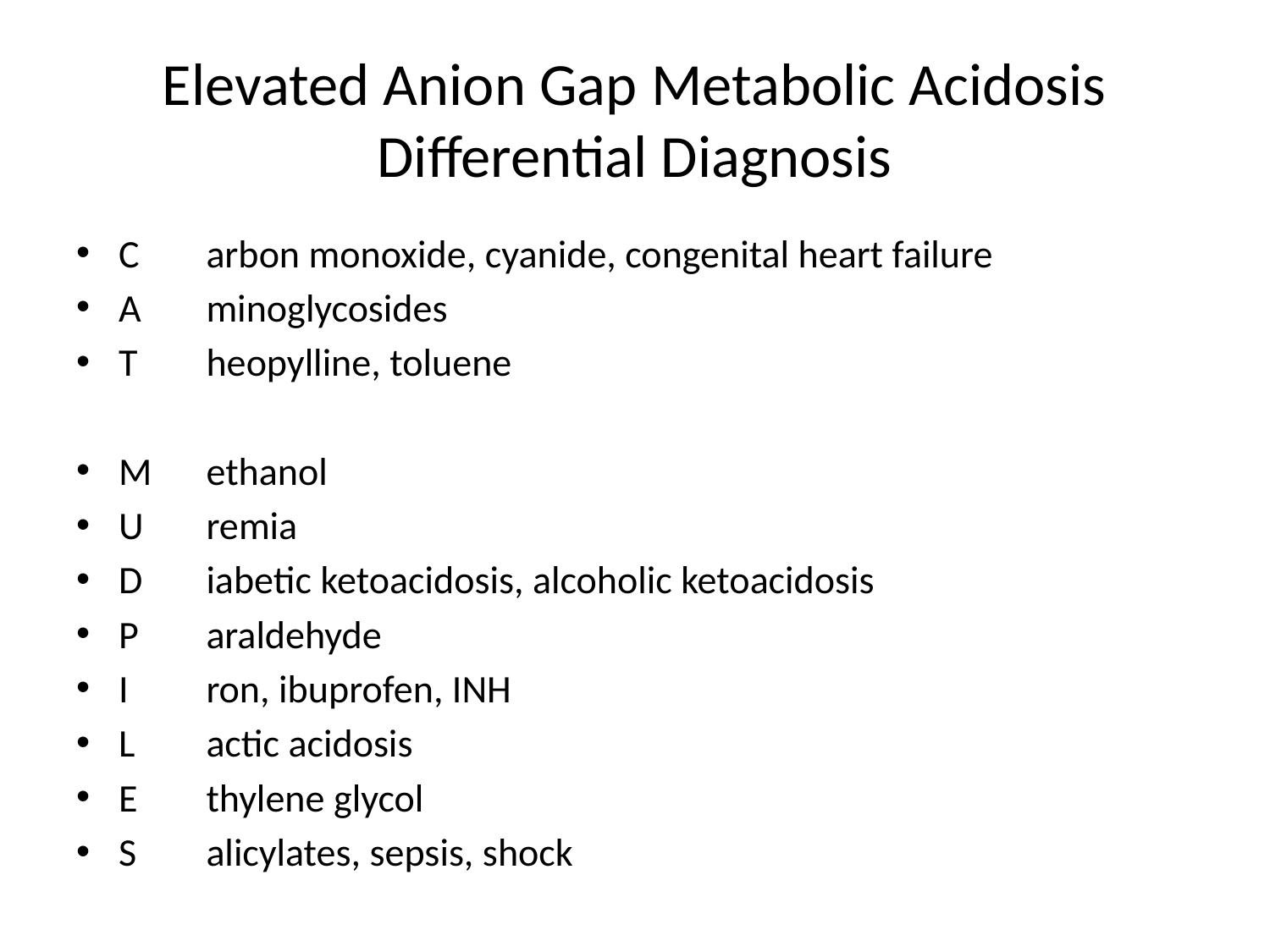

# Elevated Anion Gap Metabolic Acidosis Differential Diagnosis
C	arbon monoxide, cyanide, congenital heart failure
A	minoglycosides
T	heopylline, toluene
M	ethanol
U	remia
D	iabetic ketoacidosis, alcoholic ketoacidosis
P	araldehyde
I	ron, ibuprofen, INH
L	actic acidosis
E	thylene glycol
S	alicylates, sepsis, shock

## Slide 13
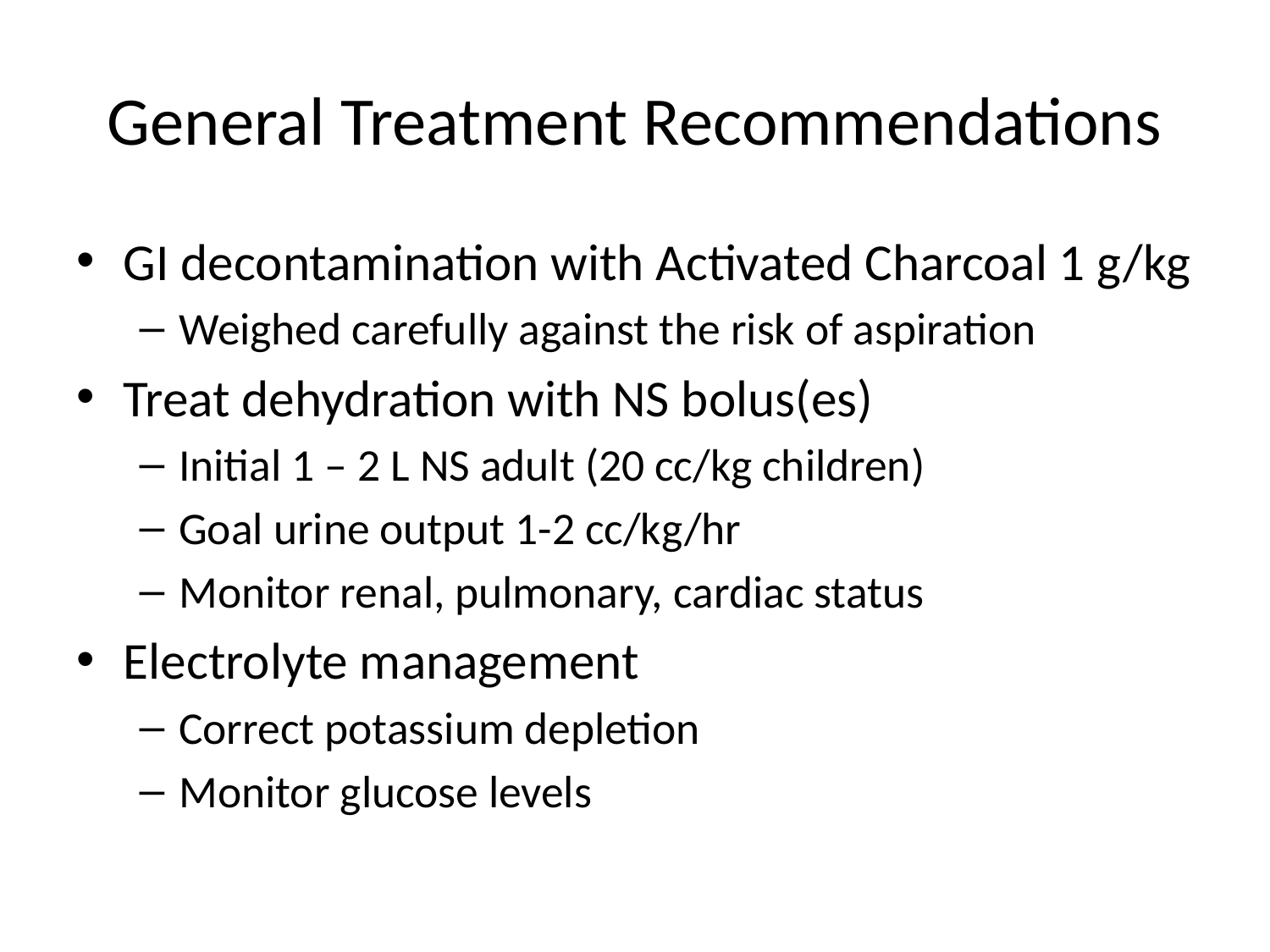

# General Treatment Recommendations
GI decontamination with Activated Charcoal 1 g/kg
Weighed carefully against the risk of aspiration
Treat dehydration with NS bolus(es)
Initial 1 – 2 L NS adult (20 cc/kg children)
Goal urine output 1-2 cc/kg/hr
Monitor renal, pulmonary, cardiac status
Electrolyte management
Correct potassium depletion
Monitor glucose levels

## Slide 14
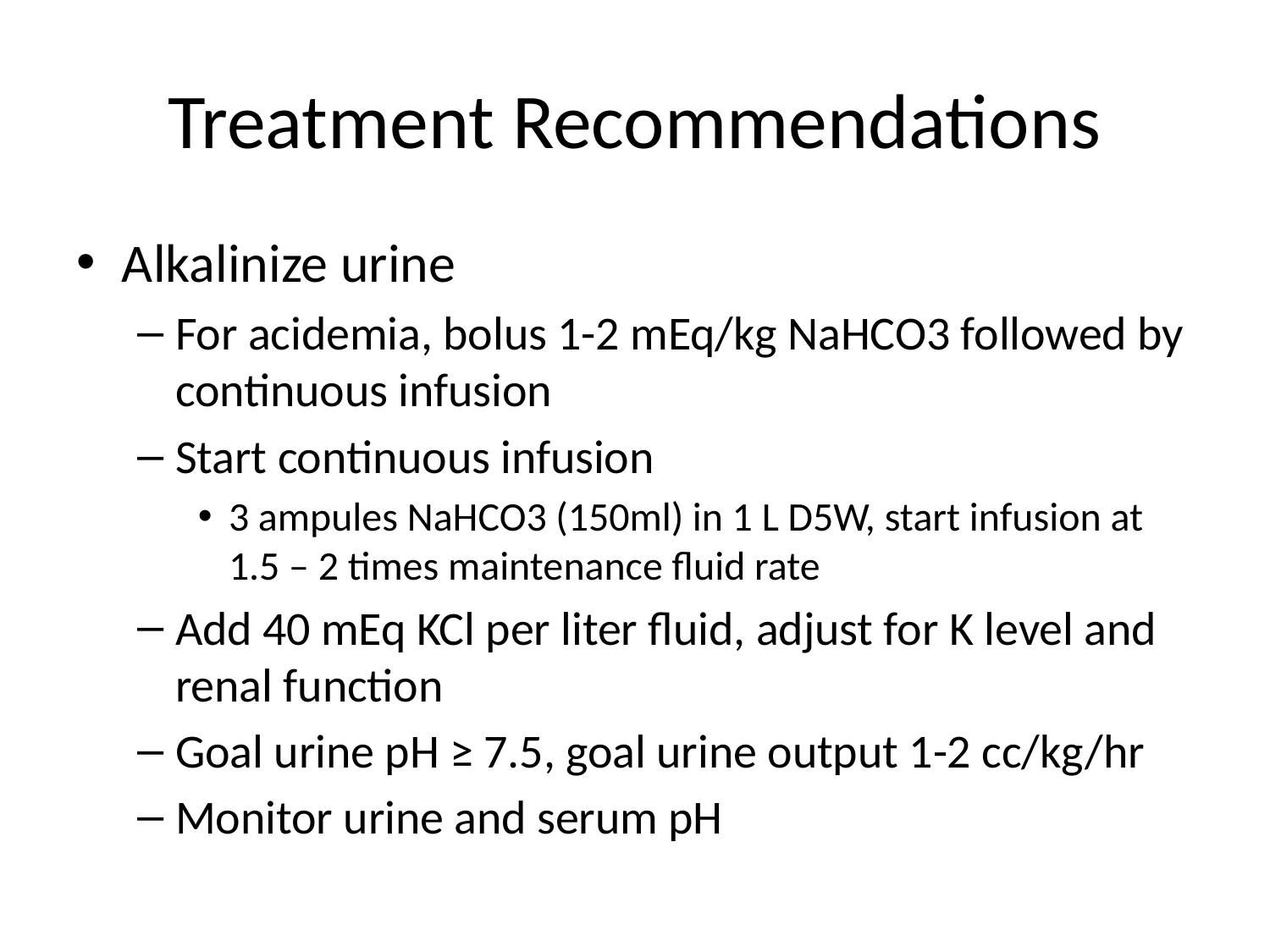

# Treatment Recommendations
Alkalinize urine
For acidemia, bolus 1-2 mEq/kg NaHCO3 followed by continuous infusion
Start continuous infusion
3 ampules NaHCO3 (150ml) in 1 L D5W, start infusion at 1.5 – 2 times maintenance fluid rate
Add 40 mEq KCl per liter fluid, adjust for K level and renal function
Goal urine pH ≥ 7.5, goal urine output 1-2 cc/kg/hr
Monitor urine and serum pH

## Slide 15
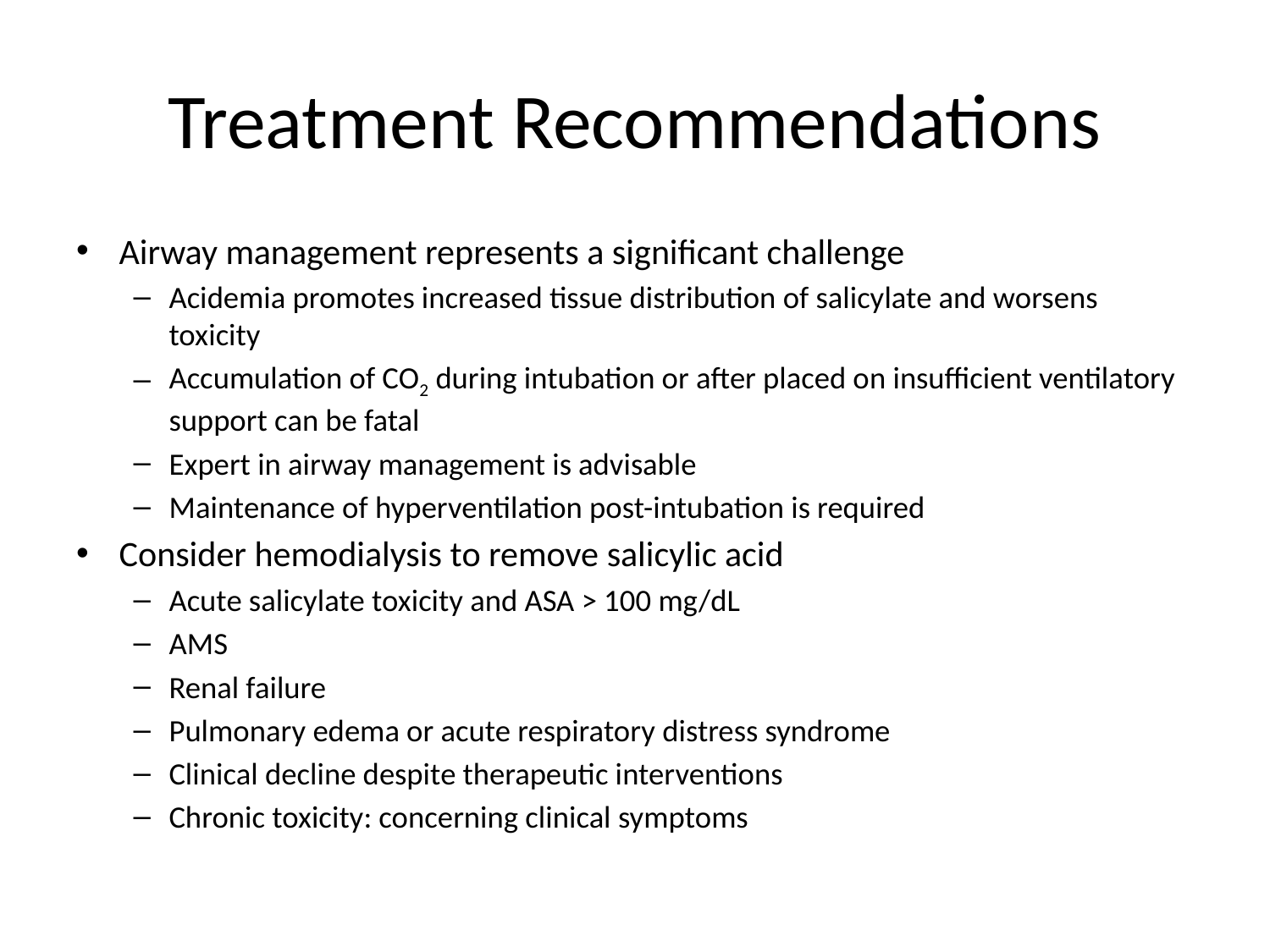

# Treatment Recommendations
Airway management represents a significant challenge
Acidemia promotes increased tissue distribution of salicylate and worsens toxicity
Accumulation of CO2 during intubation or after placed on insufficient ventilatory support can be fatal
Expert in airway management is advisable
Maintenance of hyperventilation post-intubation is required
Consider hemodialysis to remove salicylic acid
Acute salicylate toxicity and ASA > 100 mg/dL
AMS
Renal failure
Pulmonary edema or acute respiratory distress syndrome
Clinical decline despite therapeutic interventions
Chronic toxicity: concerning clinical symptoms

## Slide 16
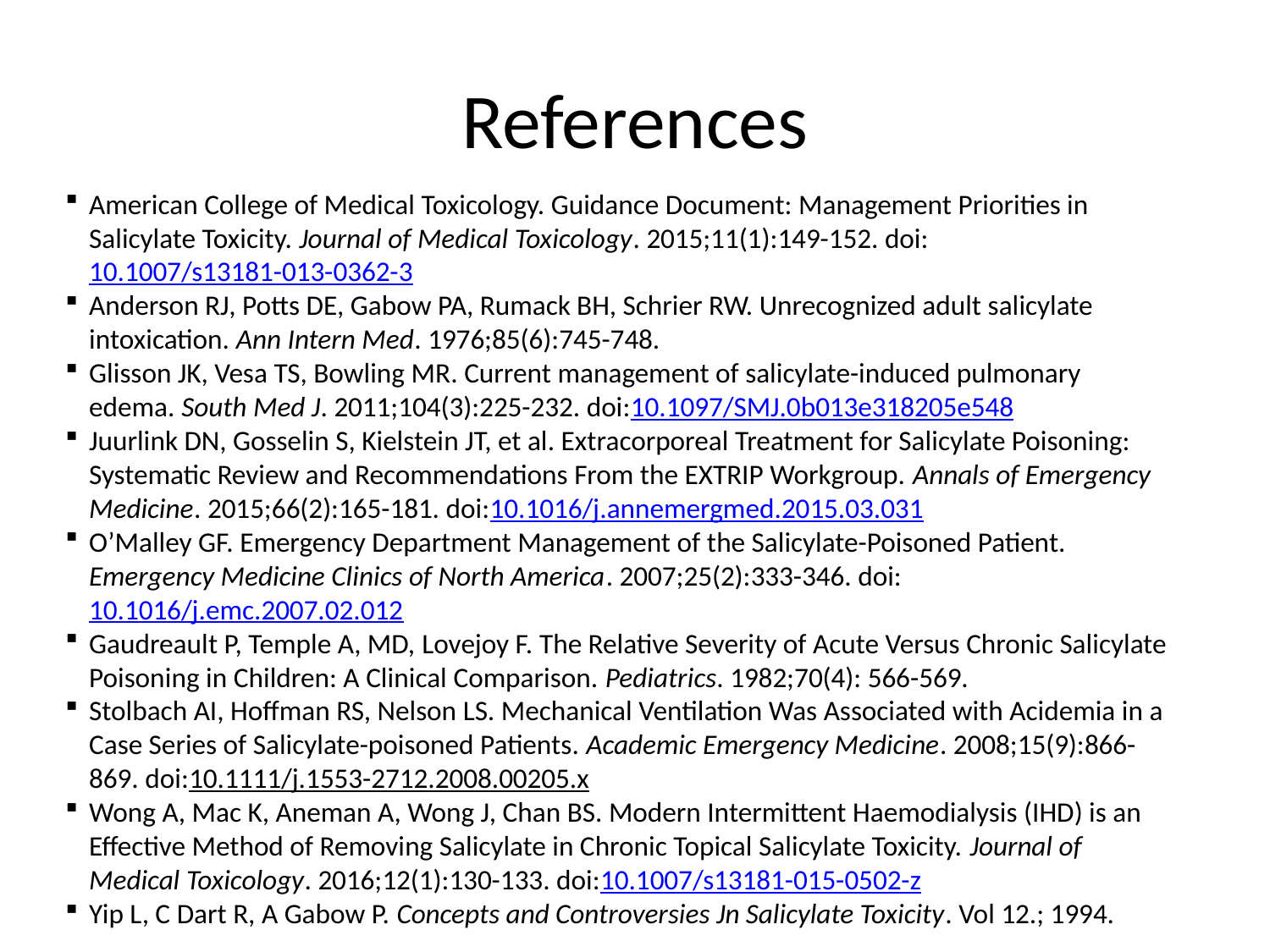

# References
American College of Medical Toxicology. Guidance Document: Management Priorities in Salicylate Toxicity. Journal of Medical Toxicology. 2015;11(1):149-152. doi:10.1007/s13181-013-0362-3
Anderson RJ, Potts DE, Gabow PA, Rumack BH, Schrier RW. Unrecognized adult salicylate intoxication. Ann Intern Med. 1976;85(6):745-748.
Glisson JK, Vesa TS, Bowling MR. Current management of salicylate-induced pulmonary edema. South Med J. 2011;104(3):225-232. doi:10.1097/SMJ.0b013e318205e548
Juurlink DN, Gosselin S, Kielstein JT, et al. Extracorporeal Treatment for Salicylate Poisoning: Systematic Review and Recommendations From the EXTRIP Workgroup. Annals of Emergency Medicine. 2015;66(2):165-181. doi:10.1016/j.annemergmed.2015.03.031
O’Malley GF. Emergency Department Management of the Salicylate-Poisoned Patient. Emergency Medicine Clinics of North America. 2007;25(2):333-346. doi:10.1016/j.emc.2007.02.012
Gaudreault P, Temple A, MD, Lovejoy F. The Relative Severity of Acute Versus Chronic Salicylate Poisoning in Children: A Clinical Comparison. Pediatrics. 1982;70(4): 566-569.
Stolbach AI, Hoffman RS, Nelson LS. Mechanical Ventilation Was Associated with Acidemia in a Case Series of Salicylate-poisoned Patients. Academic Emergency Medicine. 2008;15(9):866-869. doi:10.1111/j.1553-2712.2008.00205.x
Wong A, Mac K, Aneman A, Wong J, Chan BS. Modern Intermittent Haemodialysis (IHD) is an Effective Method of Removing Salicylate in Chronic Topical Salicylate Toxicity. Journal of Medical Toxicology. 2016;12(1):130-133. doi:10.1007/s13181-015-0502-z
Yip L, C Dart R, A Gabow P. Concepts and Controversies Jn Salicylate Toxicity. Vol 12.; 1994.
