# Supplementary material for: Chronic Salicylate Toxicity Simulation
Source: MedEdPORTAL. 2018 Aug 17;14:10741. doi: 10.15766/mep_2374-8265.10741 (PMC6342349; doi:10.15766/mep_2374-8265.10741)
Supplement: Supplementary file 1 — A. Chronic Salicylate Toxicity Simulation Case.docx B. Chronic Salicylate Toxicity Supplemental Case Materials.ppt C. Chronic Salicylate Toxicity Questionnaire.docx D. Chronic Salicylate Toxicity Debrief.pptx E. Chronic Salicylate Toxicity Evaluation Form.doc F. Chronic Salicylate Toxicity Test.docx [file mep-14-10741-s001.zip › F._Chronic_Salicylate_Toxicity_Test.docx]

Chronic Salicylate Toxicity Test

Which acid-base abnormalities are often present in a patient with salicylate poisoning?

1. Respiratory acidosis and metabolic acidosis
2. Respiratory alkalosis and metabolic acidosis
3. Respiratory acidosis and metabolic alkalosis
4. Respiratory alkalosis and metabolic alkalosis

Which of the following statements regarding salicylate induced acute lung injury is correct?

1. The etiology is related to salicylate induce renal impairment
2. This presents with serum salicylate concentrations above 65 mg/dL
3. This occurs more frequently in patients with cardiac disease, particularly congestive heart failure
4. This is most frequently observed in patients with chronic salicylate poisoning

Chronic salicylate poisoning clinically differs from acute salicylate poisoning in which of the following ways?

1. Only chronic poisoning causes neurologic abnormalities
2. Patients with chronic poisoning often exhibit signs and symptoms at lower serum salicylate levels than acute poisonings
3. Chronic poisoning does not cause respiratory alkalosis and metabolic acidosis
4. There is no utility for sodium bicarbonate in chronic salicylate poisoning

Why is it important to consider the patient’s acid-base status when interpret salicylate concentrations?

1. Acidemia favors the non-ionized state of salicylate, thus increasing it’s total body burden despite a lower serum salicylate concentration
2. Urine alkalization decreases renal elimination
3. The accuracy of the salicylate assay is affected by pH changes
4. As the serum pH increases, more salicylate enters the tissue compartments, particularly the brain

A treatment modality used to enhance renal elimination of salicylate poisoning includes which of the following?

1. Forced diuresis
2. Sodium bicarbonate therapy
3. Intubation with hyperventilation
4. Whole bowel irrigation

Which of the following is NOT an indication for hemodialysis in salicylate poisoning?

1. Altered mental status
2. High (100mg/dL) salicylate concentrations regardless of the blood pH
3. Renal failure
4. Acute lung injury
5. Tinnitus
