# Supplementary material for: Chronic Salicylate Toxicity Simulation
Source: MedEdPORTAL. 2018 Aug 17;14:10741. doi: 10.15766/mep_2374-8265.10741 (PMC6342349; doi:10.15766/mep_2374-8265.10741)
Supplement: Supplementary file 1 — A. Chronic Salicylate Toxicity Simulation Case.docx B. Chronic Salicylate Toxicity Supplemental Case Materials.ppt C. Chronic Salicylate Toxicity Questionnaire.docx D. Chronic Salicylate Toxicity Debrief.pptx E. Chronic Salicylate Toxicity Evaluation Form.doc F. Chronic Salicylate Toxicity Test.docx [file mep-14-10741-s001.zip › _Educational_Summary_Report_10741.pdf]

MedEdPORTAL®
The Journal of Teaching and Learning Resources

Crossref

Original Publication

OPEN ACCESS

Chronic Salicylate Toxicity Simulation

Mary Wittler, MD*, David A. Masneri, DO, Jennifer Hannum, MD
*Corresponding author: mwittler@wakehealth.edu

Citation: Wittler M, Masneri DA, Hannum J. Chronic salicylate toxicity simulation. *MedEdPORTAL*. 2018;14:10741. https://doi.org/10.15766/mep_2374-8265.10741

Copyright: © 2018 Wittler et al. This is an open-access article distributed under the terms of the Creative Commons Attribution license.

Appendices

A. Chronic Salicylate Toxicity Simulation Case.docx
B. Chronic Salicylate Toxicity Supplemental Case Materials.ppt
C. Chronic Salicylate Toxicity Questionnaire.docx
D. Chronic Salicylate Toxicity Debrief.pptx
E. Chronic Salicylate Toxicity Evaluation Form.doc
F. Chronic Salicylate Toxicity Test.docx

All appendices are peer reviewed as integral parts of the Original Publication.

Abstract

Introduction: Chronic salicylate toxicity is an uncommon, potentially life-threatening poisoning that requires high clinical suspicion in order to make the diagnosis. We created a simulation case that challenges learners to analyze case information, construct a differential diagnosis of an elevated anion gap metabolic acidosis with respiratory alkalosis, and initiate treatment for this toxicity. **Methods:** The simulation case was designed for emergency medicine residents and pediatric emergency medicine fellows. The activity began with a brief overview of the monitors, equipment, and simulation experience. For interns, a team of two learners comanaged the case; for senior learners, the case was managed solo. The learners had 15 minutes to complete a focused history and physical exam, request and interpret labs and studies, and initiate specific treatments. The simulation was followed by a 15-minute facilitated debrief session that included an overview of key learning points and learner performance based on an evaluation checklist. **Results:** Residents completed a postparticipation questionnaire consisting of six questions rated on a 5-point Likert scale. Overall, residents reported a high degree of satisfaction with the simulation experience. The case and debrief were effective in meeting the educational objectives and proved to be an effective modality to fill this educational gap. **Discussion:** This simulation exercise was effective in showing residents the uncommon presentation of chronic salicylate toxicity. Learners reported increased confidence in recognizing and managing this ingestion. The simulation experience closed an identified education gap and provided an experiential learning opportunity that accomplished the targeted learning objectives.

Keywords

Chronic Aspirin Toxicity, Chronic Salicylate Toxicity

Educational Objectives

By the end of this session, learners will be able to:

1. Identify the signs and symptoms of chronic salicylate toxicity in a simulated case.
2. Interpret the arterial blood gas and discriminate the causes of an elevated anion gap metabolic acidosis.
3. Describe the limitations of salicylate concentrations in chronic salicylate toxicity.
4. Identify the need for fluid resuscitation for this type of ingestion and the limitations in this simulation.
5. Illustrate the utility of sodium bicarbonate administration to alkalinize the urine in this toxicity.
6. Implement management goals for this poisoning and explain specific indications for management options.

Introduction

Chronic salicylate toxicity is a potentially life-threatening toxicity associated with delayed diagnosis and significant mortality. Compared to acute salicylate toxicity, chronic salicylate toxicity results from repeated overmedication that occurs across several days and has a gradual onset of symptoms. As such, chronic toxicity is more likely to be misdiagnosed and/or delayed in diagnosis and has high morbidity and mortality.[1,2] Compared to other pharmaceutical products, salicylate products are an uncommon cause of mortality. In the 2016 annual report of the American Association of Poison Control Centers' National Poison Data System, salicylate toxicity accounted for 36 of the total 1,492 reported fatalities.[3] To prevent mortality, salicylate toxicity requires prompt recognition and treatment. For chronic toxicity, this

necessitates familiarity with presenting signs and symptoms and a high level of suspicion. Despite the need for emergency medicine (EM) residents to have recognition of the case presentation and knowledge of its management, many residents at our institution have limited experience managing this type of toxicity. This need led to the development of a chronic salicylate toxicity case to incorporate into our simulation curriculum for our EM residents.

A simulation exercise was adapted from a real case to provide experiential learning to EM residents and pediatric EM fellows, with the following goals: allowing learners to analyze case information, construct a differential diagnosis of an elevated anion gap metabolic acidosis with respiratory alkalosis, recognize the signs and symptoms of toxicity based on a simulated case, and initiate the multimodal management interventions for this toxicity. The clinical clue in the simulation case that suggests chronic salicylate toxicity is the elevated anion gap metabolic acidosis with respiratory alkalosis; failure to diagnostically deliberate this clue easily leads to misdiagnosis of congestion heart failure, pneumonia, or alternative illness. This exercise reflects the real-life clinical decision-making process that occurs in the emergency department. A search of *MedEdPORTAL* revealed several acute salicylate toxicity simulation cases[4-6]; however, no case specific to chronic salicylate toxicity was found.

Methods

Development

At Wake Forest Baptist Medical Center, the EM residents (PGY 1-PGY 3) participate in a three-armed simulation curriculum consisting of High-Fidelity Pediatric Simulation, High-Fidelity Joint Trauma Simulation, and in situ Emergency Department Low-Fidelity Curriculum. All residents are required to participate in each simulation opportunity annually. This case was incorporated into the Emergency Department Low-Fidelity Curriculum. The simulation case exercise (Appendix A) rotated through this required curriculum on a repeat interval across 3 years. No specific prerequisite knowledge was needed by learners; however, most had exposure to analyzing an anion gap metabolic acidosis, as well as basic knowledge for stabilization of life-threatening illnesses. The facilitators included a board-certified medical toxicologist and the resident simulation director. Across 3 years, 30 PGY 1-PGY 3 EM residents and four pediatric EM fellows participated in this simulation case exercise.

Equipment/Environment

The simulation occurred in a dedicated space in the emergency department and used a simple mannequin and a programmable monitor to display vital signs. Available for use were heart monitor leads, blood pressure cuff, and pulse oximeter. Supplemental oxygen by nasal cannula and face mask was available. Standard resuscitative equipment, including defibrillator/pacemaker and intubation equipment, was present. All case information is included in Appendix A. All case diagnostic information, including X-ray images, EKG, and laboratory values, is listed in the supplemental case materials document (Appendix B).

Personnel

While not necessary, nursing or paramedic personnel can be embedded into the case if available. At our institution, the simulation facilitator executed orders and tracked verbalized interventions and requests for further information. The second faculty member provided case information, as well as playing the role of the patient and all consultants. If an intern was managing the case, then he/she was teamed with a second resident to help work through it. If a senior learner was managing the case, then a second learner provided clinical interventions or “phoned a friend” as needed.

Implementation

Over a period of 3 years, we used this case exercise with 30 PGY 1-PGY 3 EM residents and four pediatric EM fellows. No changes were made to the original case. We adapted cues for the facilitator to help deliver case content based on resident management of the case.

At the start of the simulation session, we briefed each team about the simulation environment and provided instructions for the simulation activity. This briefing included instruction to verbalize every request (place an IV, draw blood, etc.), as well as description of the general flow and organization of the case. We informed the teams of the 15-minutes time limit for performing a focused history and physical exam, ordering and interpreting laboratory studies, making treatment plans including disposition, and discussing the case with consultants. The residents then performed a history and examination of the patient, with the primary facilitator providing case information. The residents requested case information, ordered and interpreted tests, and initiated management options without interruption from facilitators. Requested studies (EKG and chest X-ray) were provided when requested. Laboratory results were provided at the facilitator's discretion for best timing of delivery; they were not provided before a complete history and physical exam had been performed. The following studies and labs were available if requested: EKG, chest X-ray, complete blood count, comprehensive metabolic panel, arterial blood gases, urinalysis, lactic acid level, troponin, B-type natriuretic peptide level, and serum toxicology levels (salicylate, acetaminophen).

As implemented at our institution, the approximately 12-minute simulation session required two facilitators: one faculty member to provide case information and one simulation faculty to act as a nurse/technician, update vital signs, and track interventions and requests for further information. We used a flip chart to track the learners' treatment interventions and requests for laboratory values. The main facilitator provided consultation as a medical toxicologist. For senior residents, the toxicologist confirmed antidote suggestions verbalized by the resident and provided further treatment recommendations. For junior residents, the toxicologist was more helpful in providing antidote suggestions and management decisions. The main facilitator also played the roles of an intensivist and a nephrologist for telephone consult. If the resident called either consultant prior to verbalization of the correct diagnosis and initiation of management interventions, then that consultant was unavailable until diagnosis of chronic salicylate toxicity was made.

Assessment

At the end of the learning experience, the residents voluntarily completed an anonymous, six-question questionnaire concerning the event (Appendix C). The questionnaire used a 5-point Likert scale and had an optional comment section. This questionnaire was developed using the Effective Evaluation: A Toolkit for Evaluating Presentations and Training publication.[7]

Debriefing

We cofacilitated a debrief session at the end of each simulation scenario. The components of the debrief were (1) learner self-assessment, (2) focused facilitation, and (3) directive feedback and teaching. We started the session by asking the learners to reflect on how they felt the scenario had gone. This usually resulted in a participant-led discussion that touched on many of the management questions and/or concerns pertinent to the case. All learners were asked to reflect on observed actions and offer reflections about the case. We reviewed the case management decisions and highlighted key learning points summarized in the didactic review (Appendix D). The facilitators offered formative feedback, including actions that were performed well and areas for improvement. This feedback represented a combination of witnessed actions and decisions made by the resident during the simulation, supplemented by reviewing the critical actions checklist (see the evaluation form, Appendix E). We used a whiteboard to highlight and address case teaching points.

Results

Since initiation of this case simulation, it has been successfully used with 30 EM PGY 1-PGY 3 residents and four pediatric EM fellows. As shown in the Table, the questionnaire results indicated favorable reception of the educational experience for all questions answered. This teaching event was effective in increasing the learners' recognition of signs and symptoms of chronic salicylate toxicity. Most learners

found that the exercise improved their confidence to critically evaluate causes of acid-base disturbances. The learners reported increased familiarity with treatment modalities and the indications to initiate treatment options. The majority of learners reported that the debrief was effective in reviewing the educational objectives.

Table. Chronic Salicylate Toxicity Simulation Questionnaire Responses (*n* = 30)

Statement	Strongly Disagree	Disagree	Neutral	Agree	Strongly Agree
Overall, I am satisfied with this educational event.	0%	0%	7%	30%	63%
I am better equipped to identify the signs and symptoms of a chronic salicylate toxicity.	0%	0%	2%	45%	53%
I am more confident in my ability to critically evaluate causes of acid-base disturbances.	0%	0%	21%	47%	32%
I have better understanding of the treatment goals for this poisoning and specific indications for management options.	0%	0%	12%	43%	45%
This event enhanced my knowledge of salicylate toxicity.	0%	0%	6%	42%	52%
The debrief was effective in presenting the educational objectives.	0%	0%	8%	49%	43%

Additional unsolicited comments included the following:

- "Thank you for running this simulation, I would have totally missed this working in the ED."
- "I suspect I have missed this."
- "Great simulation-based approach with questions and discussion."
- "I am more aware of the signs to look for after ingestion of salicylate and am more comfortable with the management."
- "Will not solely rely lab level to dictate management."

Discussion

This simulation exercise was effective in showing residents a chronic salicylate poisoning case and provided experiential learning during the deliberate analysis of the case presentation and implementation of a care pathway. All learners found value in the educational experience, based on questionnaire data. The overwhelmingly positive reception of the simulation event, as supported by the questionnaire results, indicate that this simulation event was an appropriate modality to achieve our stated learning objectives. We believe the simulation case closed a learning gap identified at our institution by providing an experiential learning opportunity to challenge the learner's knowledge base and management decision for chronic salicylate toxicity.

The case was adapted from a real case, and as such, the laboratory values and case information are realistic. In particular, this case allows for a discussion of the treatment options and the limitations of using such options secondary to the complexity of the case. This simulation was specifically designed for use with EM residents and pediatric EM fellows; however, other learners, including pediatric and internal medicine residents and critical care fellows, could use the case. We performed the simulation in the emergency department with an adult-size mannequin and a portable, programmable monitor to display vital signs. However, the case could be run in a simulation center. The case simulation was conducted using two faculty facilitators, including a medical toxicologist for case and content delivery and the EM resident simulation director to assist with interventions. Roles can vary depending on the number of participants in the simulation session and on facilitator availability. The simulation can be implemented using a single participant (or teams) and one facilitator/operator who can provide oral feedback and play additional historical or consultant roles. Using physical actors such as nursing or medic providers can enhance realism but is not required to successfully implement this case. The debrief materials and case content include references for delivery by other faculty; successful reproducibility should be obtainable. We used a single whiteboard to track interventions and requests for information during the case, as well to discuss debrief objectives. However, other means (e.g., PowerPoint) of delivering teaching content are also an option.

In addition to its utilization in this scenario, the case content has been adapted for use as an oral board test case, for one-on-one teaching, and as a case example for problem-based learning discussion (PBLD). The case can be easily adapted for an oral board review case by simply pasting the labs and studies on single pages to provide to the learner when queried. Additionally, the critical actions checklist can be adapted from formative to summative feedback without changing the critical interventions. Otherwise, all case content needed to deliver the case is present in the case summary. For use in a PBLD, the case content can be delivered to the learners, utilizing the debrief materials to direct a discussion about the metabolism, pathophysiology, presentation, and management interventions.

Barriers to implementation include the low-fidelity nature of our equipment setup. We performed the simulation within the physical space of the emergency department to help minimize the gap between the simulation experience and training environment. Learners who had not been through the simulation environment needed coaching. Orientation to the simulation equipment helped overcome this limitation. Another implementation challenge unique to this case is the rarity of the case matter. We found that junior residents were less likely to troubleshoot the elevated anion gap metabolic acidosis with the addition of other laboratory studies (lactic acid level, consideration of ketosis, and addition of acetylsalicylic acid level) as a means of narrowing the differential diagnosis. Senior residents, knowing that the medical toxicology facilitator would run a toxicology case, usually deliberated over laboratory information until the diagnosis was made. Many residents failed to make the appropriate diagnosis and required guidance. In learner reflection, most of the residents stated that they would have missed the diagnosis of chronic salicylate toxicity.

A limitation of our outcome measures is the subjective nature of our questionnaire. Because of the timing of this simulation event, we did not administer a pretest for knowledge, as it would have defeated the purpose of managing an unknown case. In the future, we could administer a posttest for knowledge (Appendix F). However, unless this is coupled with a pretest, it will not reflect knowledge gained through the simulation experience. Finally, because of the infrequency of this presentation in the emergency department, it would be extremely difficult to measure change of behavior or transfer of knowledge to the clinical setting by use of this simulation.

Overall, residents reported that the learning experience was beneficial, and this simulation case filled an educational gap identified in our residency education. The case has been incorporated into a practice case for oral board testing and is a representative case for use in the EM resident toxicology curriculum as a PBLD. As this presentation is easy to misdiagnose, our hope is that this experiential learning process translates to recognition of diagnosis in the emergency department.

Mary Wittler, MD: Assistant Professor, Department of Emergency Medicine, Wake Forest Baptist Medical Center

David A. Masneri, DO: Assistant Professor, Department of Emergency Medicine, Wake Forest Baptist Medical Center; Emergency Medicine Simulation Director, Wake Forest Baptist Medical Center

Jennifer Hannum, MD: Assistant Professor, Department of Emergency Medicine, Wake Forest Baptist Medical Center

Disclosures
None to report.

Funding/Support
None to report.

Ethical Approval
Reported as not applicable.

References

1. Yip L, Dart RC, Gabow PA. Concepts and controversies in salicylate toxicity. *Emerg Med Clin North Am*. 1994;12(2):351-364.
2. Anderson RJ, Potts DE, Gabow PA, Rumack BH, Schrier RW. Unrecognized adult salicylate intoxication. *Ann Intern Med*. 1976;85(6):745-748. https://doi.org/10.7326/0003-4819-85-6-745
3. Gummin DD, Mowry JB, Spyker DA, Brooks DE, Fraser MO, Banner W. 2016 annual report of the American Association of Poison Control Centers' National Poison Data System (NPDS): 34th annual report. *Clin Toxicol (Phila)*. 2017;55(10):1072-1254. https://doi.org/10.1080/15563650.2017.1388087
4. Mitzman J, Wittler M. Toxic ingestion: anion gap metabolic acidosis caused by acute salicylate ingestion. *MedEdPORTAL*. 2014;10:9893. https://doi.org/10.15766/mep_2374-8265.9893
5. Burns R, Reid J, Mazor S. Pediatric Toxidrome Simulation Curriculum: salicylate toxidrome. *MedEdPORTAL*. 2014;10:9913. https://doi.org/10.15766/mep_2374-8265.9913
6. Lammers RL, Sheakley ML, Hendren S. A simulated case of acute salicylate toxicity from an intentional overdose. *MedEdPORTAL*. 2018;14:10678. https://doi.org/10.15766/mep_2374-8265.10678
7. Effective evaluation: a toolkit for evaluating presentations and trainings. Northwest Center for Public Health Practice website. http://www.nwcphp.org/training/opportunities/toolkits-guides/effective-evaluation-a-toolkit-for-evaluating-presentations-and-trainings. Accessed March 17, 2018.

Received: March 20, 2018 | **Accepted:** July 26, 2018 | **Published:** August 17, 2018
