# Supplementary material for: Chronic Salicylate Toxicity Simulation
Source: MedEdPORTAL. 2018 Aug 17;14:10741. doi: 10.15766/mep_2374-8265.10741 (PMC6342349; doi:10.15766/mep_2374-8265.10741)
Supplement: Supplementary file 1 — A. Chronic Salicylate Toxicity Simulation Case.docx B. Chronic Salicylate Toxicity Supplemental Case Materials.ppt C. Chronic Salicylate Toxicity Questionnaire.docx D. Chronic Salicylate Toxicity Debrief.pptx E. Chronic Salicylate Toxicity Evaluation Form.doc F. Chronic Salicylate Toxicity Test.docx [file mep-14-10741-s001.zip › E._Chronic_Salicylate_Toxicity_Evaluation_Form.doc]

Participant’s name:

# Chronic Salicylate Toxicity

# Evaluation

# Novice Apprentice Practitioner

Communication present between team members

Appropriate division of tasks

Case Evaluation

Perform a focused history and physical exam

Consider resuscitation with isotonic fluids

Notes:

Troubleshoot acid base disorder

Consider initiation of sodium bicarbonate infusion

Acknowledging need for Nephrology consultation for dialysis

Appropriate disposition to ICU

Comments:
